# Molecular architecture of the glycogen- committed PP1/PTG holoenzyme

**DOI:** 10.1038/s41467-022-33693-z

**Published:** 2022-10-19

**Authors:** Marta Stefania Semrau, Gabriele Giachin, Sonia Covaceuszach, Alberto Cassetta, Nicola Demitri, Paola Storici, Graziano Lolli

**Affiliations:** 1 Department of Cellular, Computational and Integrative Biology - CIBio, University of Trento, via Sommarive 9, 38123 Povo, Trento Italy; 2 Protein Facility, Elettra Sincrotrone Trieste S.C.p.A, SS 14 - km 163,5 in AREA Science Park, 34149 Basovizza, Trieste Italy; 3 Department of Chemical Sciences (DiSC), University of Padua, via F. Marzolo 1, 35131 Padova, Italy; 4 Institute of Crystallography - C.N.R.- Trieste Outstation, Area Science Park – Basovizza, S.S.14 - Km. 163.5, I-34149 Trieste, Italy; 5 XRD2 Beamline, Elettra Sincrotrone Trieste S.C.p.A, SS 14 - km 163,5 in AREA Science Park, 34149 Basovizza, Trieste Italy

**Keywords:** X-ray crystallography, Intracellular signalling peptides and proteins, SAXS, Epilepsy

## Abstract

The delicate alternation between glycogen synthesis and degradation is governed by the interplay between key regulatory enzymes altering the activity of glycogen synthase and phosphorylase. Among these, the PP1 phosphatase promotes glycogenesis while inhibiting glycogenolysis. PP1 is, however, a master regulator of a variety of cellular processes, being conveniently directed to each of them by scaffolding subunits. PTG, Protein Targeting to Glycogen, addresses PP1 action to glycogen granules. In Lafora disease, the most aggressive pediatric epilepsy, genetic alterations leading to PTG accumulation cause the deposition of insoluble polyglucosans in neurons. Here, we report the crystallographic structure of the ternary complex PP1/PTG/carbohydrate. We further refine the mechanism of the PTG-mediated PP1 recruitment to glycogen by identifying i) an unusual combination of recruitment sites, ii) their contributions to the overall binding affinity, and iii) the conformational heterogeneity of this complex by in solution SAXS analyses.

## Abstract (web summary)

Glycogen metabolism is tightly regulated. Here the authors describe the 3D structure of the PP1/PTG protein complex, which plays a prominent role in the activation of glycogen synthesis and in the pathogenesis of Lafora disease, the most severe form of pediatric progressive epilepsy.

## Introduction

Glycogen metabolism is tightly regulated through tissue-specific mechanisms responding to differentiated missions, i.e., blood glucose homeostasis in the liver and mechanical work in muscles, among others. Different organ-restricted isozymes participate in glycogen metabolism, being variably regulated allosterically and post-translationally^1^. Glycogen synthases (GYSs) and glycogen phosphorylases (PYGs) are inversely regulated by phosphorylation, promoting glucose mobilization through PYGs while inhibiting GYSs. Dephosphorylation of both enzymes is exerted by protein phosphatase 1 (PP1), a pleiotropic phosphatase involved in the regulation of cell growth and protein synthesis, other than glycogen metabolism. PP1 is directed to its different pathway-specific substrates by scaffold proteins; PPP1R3 are those orienting PP1 towards GYSs and PYGs.

The human genome encodes for seven PPP1R3 (PP1 regulatory subunit 3) proteins, none sharing > 40% aminoacidic identity to any of the others. Nonetheless, each of them is very well conserved among mammals and has different tissue distribution, suggesting specific and non-redundant functions^2^. PPP1R3A, aka GM, only expressed in skeletal muscles and heart, is a large protein of 1122 amino acids, while PPP1R3B (285 residues), alias GL, is the hepatic glycogen-targeting PP1 regulatory subunit; both are themselves regulated by phosphorylation. PPP1R3C (317 amino acids), also named protein targeting to glycogen (PTG), is ubiquitously expressed. The other four PPP1R3 proteins appear to be minor isoforms with limited expression although differentiated in the various organs.

Glycogen is stored in minimal amounts in neurons while relatively abundant in astrocytes, where glucose is mobilized to support energy demand during neurotransmission, provide neuroprotection under glucose deprivation and spare glucose to neurons^3^. PTG has a very relevant role in glycogen metabolism in the brain, where its levels are post-translationally regulated^4^. The malin protein is a ubiquitin ligase directing PTG to proteasomal degradation^5^. PTG ubiquitination by malin requires the assembly of a ternary complex also comprising the laforin phosphatase. Mutations in malin or laforin cause PTG accumulation, promoting glycogen synthesis in neurons by directing PP1 to GYS and PYG^6,7^. This results in the appearance of neurotoxic inclusion bodies formed by insoluble polyglucosans called Lafora bodies (LB), which are ultimately responsible for Lafora disease (LD)^8,9^.

LD is the most severe form of teenage-onset progressive epilepsy. Seizures increase in frequency accompanied by cognitive decline. Affected individuals usually die within ten years of onset, in a vegetative state and constant myoclonus. No therapy is available and the treatment is defined on the patient’s signs and symptoms, mainly helping with seizures managing. The reduction of glycogen production, obtained by knocking out PTG in LD mice, resulted in a nearly complete LB disappearance and resolution of neurodegeneration and myoclonic epilepsy^10,11^. Importantly, skeletal muscles of PTG knockout mice still make glycogen, 70% of normal, avoiding the cardiomyopathy associated with low activity GYS, as observed in glycogenosis type 0. These findings indicate that small molecules affecting PTG function/levels emerge as a promising therapeutic strategy for LD. In particular, a PTG-directed PROTAC (Proteolysis Targeting Chimera) could restore the physiological PTG homeostasis by inducing its ubiquitination and degradation. Up to date, there was no structural data of PTG and PTG/PP1 complex which would allow for identification of potential druggable pockets.

In this work, we present a comprehensive structural analysis of PTG and its complex with PP1 and carbohydrates both *in cristallo* and *in solution*, together with a detailed evaluation of the most relevant regions involved in complex formation. While shedding light on the mechanism by which PTG recruits PP1 to glycogen, this work also poses the basis for developing PTG-interfering small molecules that could be of therapeutic relevance for LD.

## Results

### Crystal structure of PTG CBM21 in complex with β-cyclodextrin

**Fig. 1:**
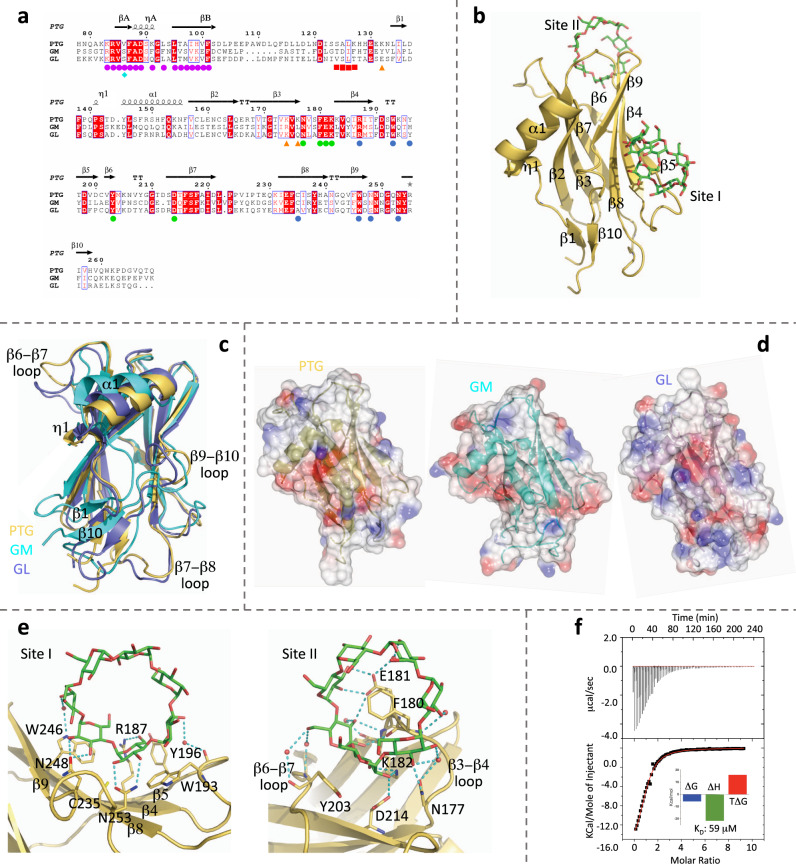
PTG binding mode to carbohydrates. **a** Structural alignment for PTG, GM, and GL. PTG residues interacting with β-cyclodextrin are indicated with blue circles for site I and green circles for site II. PTG ubiquitinated lysines are highlighted with orange triangles, while phosphorylated serine in GM and GL with a cyan diamond. Magenta circles are shown below PP1-interacting residues from the PTG N-terminal region (RVXL-containing signature), while red squares point to the SALK signature. **b** Crystallographic structure of the PTG CBM21 (yellow) in complex with β-cyclodextrin (green). **c** Superposition of CBM21 structures of PTG (yellow), GM (cyan), and GL (purple). **d** Electrostatic surface representation for the three CBM21. **e** Detailed interaction with β-cyclodextrin at the two binding sites. **f** ITC returns an enthalpically-driven binding of β-cyclodextrin to the CBM21 with a 1:1 stoichiometry.

PTG is composed of 317 amino acids with a structured central region, containing a carbohydrate-binding module 21 (CBM21, residues 133–261), flanked by largely disordered regions. We determined the crystal structure of the PTG CBM21 in complex with β-cyclodextrin in two different space groups at 1.5 and 2.0 Å resolution. The domain is organized in a β-sandwich of eight antiparallel strands (β2-β9) divided into two β-sheets, with an overall immunoglobulin-like fold (Fig. 1a, b). On one side of the sandwich, the N-terminal region forms a short 3_10_ helix immediately followed by an α-helix and connects to the C-terminal segment through an additional two strands short β-sheet (N-ter β1 and C-ter β10). The four chains (one in the orthorhombic crystal and three in the asymmetric unit of the monoclinic system) are identical with an average rmsd of 0.36 Å (over Cα atoms). The only known CBM21 structures are those of human GL, determined by NMR in the apo form and deposited to the PDB (code 2EEF) without any accompanying publication, rabbit apo GM (NMR, PDB code 2M83)^12^, and the crystal structures of apo and holo glucoamylase CBM21 from the Rhizopus oryzae fungus (PDB 2VQ4, 2V8L, 2V8M, 4BFO, and 4BFN)^13,14^.

The PTG CBM21 structure is similar to GL with significant deviations for β6-β7 (5.4 Å distance between PTG-Y208 Cα atom and the corresponding GL-Y184) and β9-β10 loops, other than for N- and C-termini, and minor divergences for the β7-β8 loop and the helical region (Fig. 1c). Differences are instead more pronounced with respect to the GM CBM21. In the overall conservation of the eight strands β-sandwich, almost all loops deviate significantly, as well as the additional secondary elements β1, β10, and the 3_10_ η1 (missing in GM) and α1 helices, conferring to the domain a different shape (Fig. 1d). This is particularly evident for the surface composed of the β1-β10 sheet, the η1 helix with its surrounding loops and the β7-β8 loop.

In the PTG CBM21 crystal structure, the β-cyclodextrin molecule binds at the interface between symmetry-related copies occupying site I in one chain and site II in the other (Supplementary Fig. 1). In site I, half of the cyclodextrin molecule lies on the shallow tray generated by the β4-β5-β8-β9 sheet together with the β4-β5 and the β9-β10 loops (Fig. 1e). Major van der Waals contacts are with Arg187, Trp193, Tyr196, Cys235, and Trp246 together with H-bonds directed to Arg187, Asn248, and Asn253 side chains. Additional water-bridged H-bonds are formed with Trp193 main chain and Trp246 side chain. At site II (Fig. 1e), the cyclic oligosaccharide inserts in between the β3-β4 and the β6-β7 loops with a close net of H-bonds connecting it to PTG residues Asn177, Phe180, Glu181, Lys182, Asp214, and Tyr203 (this last being water-mediated); additionally, extensive van der Waals contacts are present with Lys182, Tyr203 and especially Phe180 that, inserting in the cyclodextrin ring, becomes fully buried.

The same two sugar-binding sites have been identified in the crystal structures of Rhizopus oryzae CBM21^13,14^; site II is the primary binding site, while site I has been hypothesized to contribute to the binding of long-chain soluble polysaccharides and insoluble starch or to the relaxation and unwinding of helical amylose^15,16^. Interestingly, chemical shift perturbation/titration experiments showed that GM CBM21 only binds carbohydrates at site II^12^. Analogously, we measured a *K*_D_ of 59 ± 5 μM with a 1:1 stoichiometry by ITC titration of PTG CBM21 with β-cyclodextrin (Fig. 1f, Supplementary Table 1). The presence of a second binding site cannot be unambiguously confirmed nor excluded by the ITC experiment; if present, however, the affinity of this site for β-cyclodextrin is very low (Supplementary Table 1). The observed interaction at site I configures then as a crystallographic interface (Supplementary Fig. 1) or as an ancillary carbohydrate-interacting site, which binding to glucose chains is cooperatively reinforced by the dominant interaction at site II; while site I shows some sequence divergence, all site II residues are conserved in PTG, GM and GL (Fig. 1a). In accordance with the predominantly polar nature of this interaction, β-cyclodextrin binding is dominated by the enthalpic contribution, counteracting the observed unfavorable entropic term (Fig. 1f). This last can be attributed to the freezing of the β3-β4 and β6-β7 loops, flexible in all apo CBM21 structures, while more rigid in the holo structures of PTG and *R. oryzae* glucoamylase^12–14^. The titration through grating-coupled interferometry (GCI) returned a comparable *K*_D_ of 37 ± 7 μM determined by relatively fast *k*_on_ and *k*_off_ (Supplementary Fig. 3 and Supplementary Table 2, a description of the GCI technique is reported in the methods section). By comparison, *R. oryzae* CBM21 binds β-cyclodextrin with a *K*_D_ of 5.1 μM^15^, while for the same domain in GM *K*_D_ of 8.2 and 27.6 μM have been reported for β and α-cyclodextrin, respectively^12^.

### Crystal structure of PP1 in complex with PTG N-terminal peptide

**Fig. 2:**
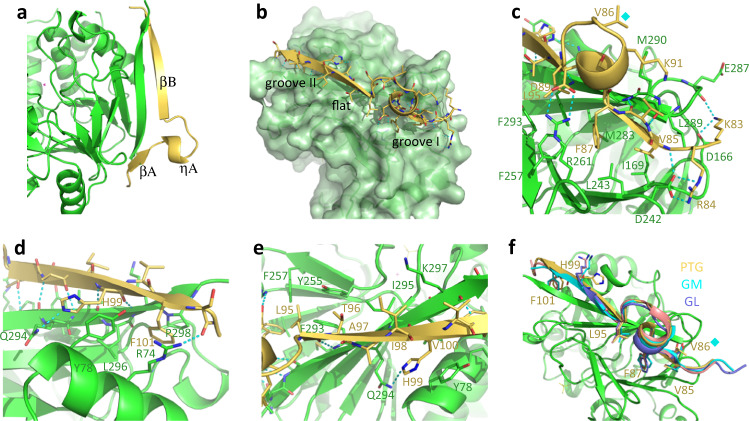
Crystal structure of PP1 in complex with the PTG peptide 81–107. **a** The PTG peptide (yellow) organizes in two β-strands (and an intervening 3_10_ helix) extending the PP1 (green) β sheet. **b** The peptide perfectly adapts to the PP1 surface filling the two grooves with hydrophobic side chains. **c** Detailed interactions of the PTG RVVF region with the first PP1 groove. Val86 substituting the GM and GL phosphorylated serine is marked with a cyan diamond. **d** Detailed interactions of the PTG peptide at the second PP1 groove. **e** Hydrophobic interactions contribute to binding of the PTG peptide to the PP1 flat region. **f** Comparison of the binding modes to PP1 (green) of RVXL-containing peptides from PTG (yellow), GM (cyan), and GL (purple). Val86 substituting the GM and GL phosphorylated serine is marked with a cyan diamond.

The PTG N-terminal region contains the PP1-binding signature RVxF (^84^RVVF^87^). PP1 was then co-crystallized with the PTG peptide 81–107, encompassing the above signature. The region 83–103 could be easily reconstructed in the electron density map (2.05 Å resolution). It organizes on the PP1 surface as two β-strands, with an intervening 3_10_ helix, pairing the PP1 C-terminal β-strand (Figs. 1a and 2a). In the context of the various intermolecular H-bonds involving the main chain atoms responsible for the β-sheet extension, side chains of the PTG peptide also significantly contribute to the binding (Fig. 2b). The aliphatic region of Lys83 is in contact with PP1 Leu289, while its terminal amino group forms a salt bridge with PP1 Asp166 side chain and an H-bond with PP1 Glu287 main chain oxygen (Fig. 2c). PTG Arg84 complements PP1 Asp242 side chain, which also receives an H-bond from the main chain nitrogen of PTG Val85. PTG Val85, Phe87, and Leu95 are almost entirely buried in the PP1 hydrophobic groove constituted by Ile169, Leu243, Tyr255, Phe257, Arg261, Met283, Leu289, and Phe293, all also becoming barely solvent-exposed (Fig. 2b, c). Following an almost flat region on the PP1 surface, a second groove accommodates PTG His99, stacking to PP1 Tyr78 and hydrogen bonding to Gln294, and PTG Phe101, sandwiched between PP1 Arg74, Leu296, and Pro298 (Fig. 2b, d). Additional interactions involve i) the PTG helical turn that allows Val86, Ala88, Lys91, and Leu93 to embrace the protruding PP1 Met290 and PTG Asp89 to complement the PP1 Arg261 side chain (Fig. 2c), and ii) PTG residues Thr96, Ala97, Ile98 and Val100 lining with the flattest part of the PP1 surface including Ile295 and the aliphatic regions of Gln294 and Lys297, again becoming largely solvent-excluded (Fig. 2e).

Comparison of this structure with those of PP1 in complex with the analogous GM and GL regions^12,17^ (5ZQV and 5ZT0 containing mouse PP1 and human GM and GL, respectively, and 6DNO with human PP1 and rabbit GM peptide) highlights the overall conserved binding mode and secondary structure organization (Fig. 2f). The PTG amino acids occupying the two PP1 grooves, largely contributing to the binding via hydrophobic and stacking interactions (Val85, Phe87, Leu95, His99, and Phe101), are all conserved except for His99 substituted by lysine in both GM and GL (Fig. 1a). In the PP1-GL structure, the PP1 second groove is not occupied by the GL Phe79 side chain (missing density from Val78 onwards), corresponding to PTG Phe101 and GM Phe80, both importantly contributing to the binding interface. It remains to be clarified whether this is a peculiarity of GL or, most probably, a crystallographic artifact due to packing restriction and poor resolution of the associated structure (3.3 Å).

In GM and GL, the X residue in the RVXF region is the serine phosphorylated following the activation of the adrenalin or glucagon signaling cascades^18–22^ (Figs. 1a, 2c, f). When glucose is requested, phosphorylation of GM and GL drives their dissociation from PP1, in turn becoming unable to act on glycogen metabolism. The same residue is a valine in PTG, instead regulated by ubiquitination on Lys132, Lys174, and Lys176 (Fig. 1a); of these lysines, only one is conserved and present only in GL, not anyway being reported as an ubiquitination site^23^.

### Crystal structure of the PP1/PTG complex

Crystals of PP1 in complex with PTG 70–264, encompassing both the PP1-binding region and the CBM21, anisotropically diffracted to 2.7 Å in two directions, but only to 3.9 Å in the third and worst direction. The structure was determined by molecular replacement using the above-described PTG-CBM and PP1-PTG(81–107) structures. Two PP1/PTG copies/asu were located with additional electron density corresponding to cyclodextrin molecules and part of the PTG sequence intervening between the N-terminal region and the CBM21.

**Fig. 3:**
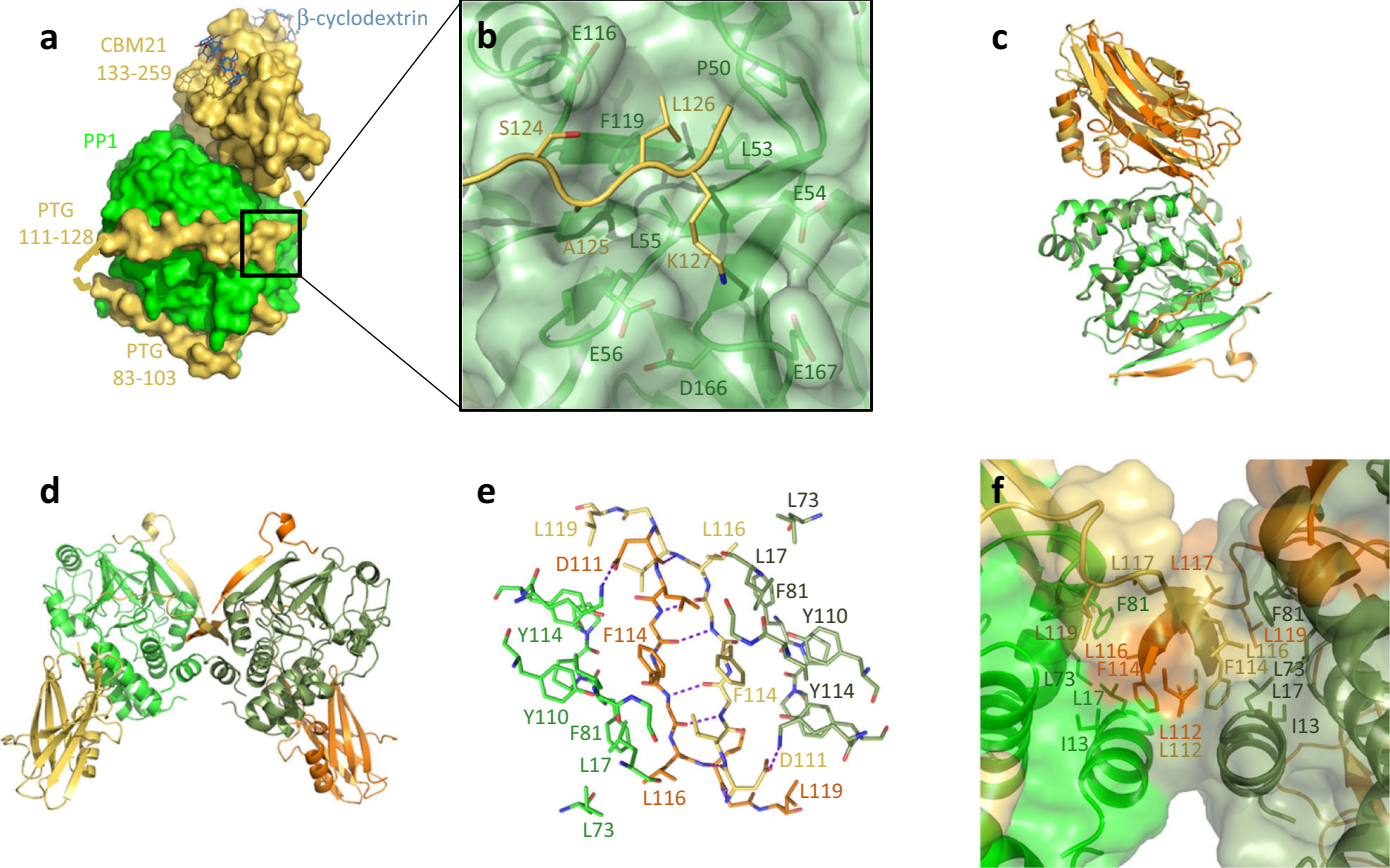
Structural organization of the ternary PP1/PTG/cyclodextrin complex. **a** The PTG (yellow) region (aa. 111–128) crosses the PP1 surface (green) to connect its N-terminal stretch to the CBM21 on the opposite PP1 side. **b** The PTG residues ^124^SALK^127^ fill a small groove on the PP1 surface. **c** Superposition of the two dimers in the asymmetric unit shows the different orientations assumed by CBM21 (yellow and orange) with respect to PP1 (light and dark green). **d** The butterfly-shaped intertwined PP1/PTG dimer. **e** β-strands pairing at the dimer interface. **f** Hydrophobic core at the dimer interface.

All previously described interactions between the CBM21 (residues 133–259) and cyclodextrin are conserved, as is the binding mode of the PTG region 83–103 to PP1. Residues 111–128 could be almost identically built in the two copies, crossing PP1 and joining the N-terminal peptide on one PP1 side with the CBM21 on the opposite PP1 surface (Fig. 3a). Extensive interactions are observed for the PTG residues ^124^SALK^127^ (Fig. 3b). Ala125 and Leu126 insert in a small groove defined by PP1 residues Pro50, Leu53, Leu55, and Phe119; the flanking Ser124 contacts PP1 Glu116, while Lys127 inserts in a very acidic region of the PP1 surface defined by Glu54, Glu56, Asp166, and Glu167.

The CBM motif sits on an almost flat PP1 surface by using its β1 strand, the following 3_10_ helix and the intervening loop. This poorly interlocked interface is indeed slightly different in the two dimers in the asymmetric unit, then pliable, due to a rotation of the CBMs in the context of identical locations for the other PTG regions (Fig. 3c). It is mainly polar, variably (depending on the orientation) involving PTG residues Ile135, Asp137, Pro139, Gln140, Thr143, and Asp144 and PP1 amino acids Leu40, Lys41, Arg43, Glu44, Leu47, Ser48, Lys147, Lys150, and Asp154 (Supplementary Fig. 2).

Interestingly, the PP1/PTG complex organizes in the crystal as a butterfly-shaped tight pseudo-knotted dimer of dimers (Fig. 3d). PTG region 111–117 pairs with its symmetric mate forming an intertwined antiparallel β-sheet (Fig. 2e, f). The driving force in this assembly, however, relies on a very hydrophobic core, formed by residues in all four chains, namely PTG Leu104, Phe106, Leu116, Leu117, and Leu119 and PP1 Ile13, Gly14, Leu17, Leu73, Phe81, Pro82, Pro83, Tyr110, and Tyr114, which become largely solvent-excluded. For the tetramer to form, PTG must detach its CBM21 (and transiently the SALK region) from the PP1 surface, thus confirming the dynamic nature of the interaction in this region, while remaining complexed to PP1 through its N-terminal region.

### Comparison with other PP1 complexes

**Fig. 4:**
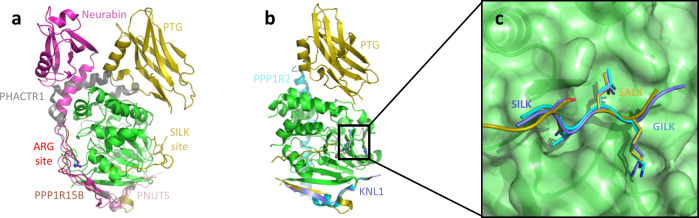
Comparison of PP1 complexes. **a** Superposition of PP1 (green) in complex with PTG (yellow), Neurabin (magenta), PHACTR1 (gray), PPP1R15B (brown), and PNUTS (pink). The SILK site in PTG locates on an opposite PP1 surface with respect to the ARG site in the other proteins. **b** Superposition of PP1 (green) in complex with PTG (yellow), KNL1 (purple), and PPP1R2 (cyan). **c** The SILK region in the three proteins perfectly superposes (color code same as above).

A limited number of PP1 structures in complex with large portions of the partner proteins are available. The identical PP1 holoenzymes with Neurabin-1 and Neurabin-2^24^ (PDB 3EGG and 3HVQ) significantly diverge from the PP1/PTG complex (Fig. 4a). The flexible region connecting the N-terminal PP1-recognition portion of Neurabin and its PDZ domain also embraces PP1 but on the opposite side with respect to the PTG linker region; the PP1/PDZ interface is adjacent but different from the PP1/CBM21. We notice that mutational studies and the absence of electron density for one PDZ domain out of the two in the asymmetric unit indicate that recognition of PP1 and Neurabin relies on the RVXL motif and surrounding residues, while the other interacting surfaces configure as transient. The path followed by the linker region of Neurabin is similarly retraced in three other PP1 holoenzymes, namely those with PHACTR1 (phosphatase and actin regulator 1), PPP1R15B and PNUTS (PP1 nuclear targeting subunit), directed by the so-called Arg motif^25–27^.

PTG linker instead perfectly superposes, using its ^124^SALK^127^ sequence, to similar stretches observed in KNL1 (kinetochore scaffold 1 protein) and PPP1R2 (protein phosphatase inhibitor 2)^28,29^ (Fig. 4b, c). This region, known as the SILK PP1-binding motif, was reported, hypothesized and always searched at the N-terminus of the RVXF motif^30–33^. This evidence of a SILK motif downstream of the RVXF binding region highlights that previous computational studies may have missed combinations of PP1-binding motifs in its targeting subunits, as was the case for PTG. Notably, the SALK sequence is not conserved in GM and GL, with poor homology among the three proteins in the linker region (Fig. 1a). Instead, it is worth noticing that the SSILK region of KNL1 (corresponding to PTG SSALK) is phosphorylated by Aurora B on both serines, thus impairing its binding to PP1, which in turn is removed from the kinetochore^28,34^. Hurley et al.^29^ showed that the lysine of the SILK sequence is the major determinant of the interaction; by locking in the very acidic region of PP1 (defined by Glu54, Glu56, Asp166, and Glu167), this lysine also directs the preceding IL residues to the small hydrophobic groove on the PP1 surface. However, the Ile to Ala substitution probably loosens the binding of the SALK sequence with respect to the consensus (G/S)IL(K/R), as suggested by the strong electron density observed for the Ile residue in the other SILK-containing PP1 complexes.

### PTG binding to PP1 is dictated by the RVXF motif with the SALK sequence, but not the CBM21, contributing to the binding affinity

GCI titrations confirmed that the main PTG binding determinant to PP1 resides in its RVVF region and surrounding residues with a *K*_D_ = 14 nM for the PTG peptide 81–107. For the extended peptide 81–132, *K*_D_ almost halved, indicating a contribution of the SALK sequence to the binding affinity, although not dramatic. On the other hand, the PTG construct 70–264 did not bind PP1 any better, and for the CBM21 aa. 132–264 no interaction was detected; the CBM21 then does not recognize PP1 via a stable interface (Supplementary Table 2 and Supplementary Fig. 3). Notably, the present structure was obtained following crystal dehydration, a procedure leading to increased resolution of diffraction data. Low-resolution structures were determined without dehydrating the crystals; in this case and similarly to what was observed in the PP1/Neurabin complexes, electron density was well defined for both the RVXF and the SALK regions in both copies in the asymmetric unit, but for only one of the CBM domains. The poor electron density for the other CBM confirms it can assume multiple orientations with respect to the PP1 surface, in the context of bound RVXF and SALK regions.

A comparison can be attempted with GM, for which similar experiments were performed^12^. The GM peptide 64–105, corresponding to PTG 84–131, binds PP1 with *K*_D_ = 114 nM, almost 15 times weaker than what was measured for PTG. The measured *K*_D_ = 21 nM for GM 64–237 allowed concluding that the CBM contributes to PP1 interaction, although binding for the isolated CBM could not be detected. Interestingly, GM residues interacting with PP1 were mapped by NMR in the region comprising the β4-β5, β7-β8, β9-β10 loops and the β10 strand. The PP1-interacting region in PTG comprises instead the β1 strand, the η1 helix, and the intervening loop. All the above-mentioned loops and secondary structure elements are those showing the major structural deviations in GM, GL, and PTG (Fig. 1c, d). Additionally, the region intervening between the N-terminal PP1-binding signature and the CBM (including the PTG SALK sequence) shows no sequence conservation in the three paralogues, being also significantly shorter in GM (24 vs. 31 aa). Based on the above NMR data, a model has been proposed for the PP1/GM complex with the GM-CBM located very differently with respect to PTG-CBM^12^. It appears that, starting from a very homologous recognition through the N-terminal RVXF segment, the overall structural organization of the following regions on the PP1 surface largely deviates in PTG and GM. While in GM the CBM orientation is only restrained by the RVXL motif, the presence in PTG of the SALK sequence reorients the correspondent CBM to an opposite location, resulting in complexes with very different shapes.

### The PTG CBM21 adopts multiple orientations in the PP1/PTG complex

**Fig. 5:**
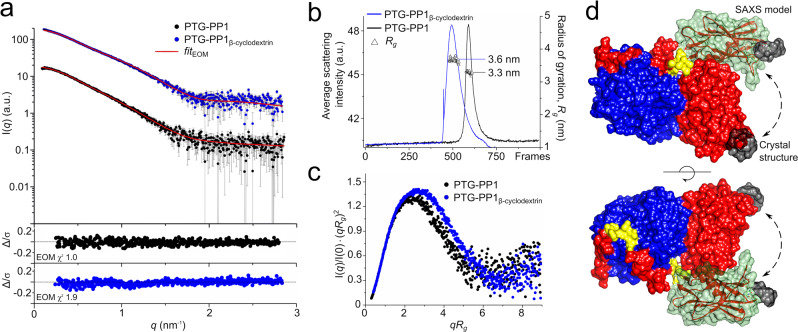
SAXS studies on the binary and ternary PTG-PP1 complexes. **a** Shown are *I*(*q*) versus *q* experimental SAXS profiles for apo PTG-PP1 (black dots) and bound to cyclodextrin (blue dots) with the EOM fit (red lines). SAXS curves were obtained through averaging of buffer-background subtracted frames across the entire elution traces of the SEC-SAXS experiments (approximately *n* = 50 frames averaged, see Supplementary Table 4). Error bars represent an estimate of the experimental error, σ, on the intensity recorded for each value of *q* assigned by data reduction software^57, 58^. Chi^2^ values (*χ*^2^) for the EOM fitting are indicated. The curves are shifted by an arbitrary offset for better comparison. The lower plots show the error-weighted residual difference plots. **b** SEC-SAXS chromatograms of PTG-PP1 complex alone and bound to β-cyclodextrin. The black and blue lines represent the total summed scattering intensity for PTG-PP1 and PTG-PP1 + β-cyclodextrin, respectively; triangles represent the calculated radius of gyration, *R*_*g*_ in nm, in the selected frames. **c** Dimensionless Kratky plots for PTG-PP1 complex alone (black circles) and bound to β-cyclodextrin (blue circles). **d** Superposition of an illustrative 3D EOM structure describing the ternary complex together with the corresponding X-ray crystal structure solved in this study. All the structures are represented in surface mode. PP1 protein is shown in blue; the PTG segments from residue 83–102 and from 111 to 128 are shown in red; missing PTG residues were modeled with EOM and shown in yellow. In red, the PTG CBM21 domain bound to β-cyclodextrin (in dark gray) in the position interacting with PP1 as observed in the crystal structure. In light green with transparency, the EOM model for PTG CBM21 domain bound to β-cyclodextrin and unbound from PP1.

In solution complementary analysis were carried out on both the binary PP1/PTG and on the ternary PP1/PTG/β-cyclodextrin complexes using integrative SAXS approaches. This technique measures the X-ray scattering pattern from a randomly oriented protein in solution to provide low-resolution structural information such as molecular mass and overall shape structural parameters. SAXS emerged as a powerful orthogonal structure validation tool for X-ray protein crystallography thanks to advances of data processing tools that allowed, for instance, the modeling of protein structures containing flexible domains^35^. In our experiments, the coupling of analytical size-exclusion chromatography (SEC) with SAXS (SEC-SAXS) on a dedicated synchrotron beamline has ensured accurate interpretation of scattering data deriving only from monodispersed elution peaks^36^. The *R*_*g*_ and I(0) traces as functions of frames show that the protein complexes were well separated in individual peaks and monodispersed. The ternary complex elutes earlier than the binary suggesting that it has a larger hydrodynamic radius (Fig. 5b). Primary data analysis from scattering curves showed that both apo and cyclodextrin-bound PTG-PP1 complexes are similar with an elongated and folded shape (Fig. 5a). The P(*r*) function calculated by indirect Fourier transformation (Supplementary Fig. 4b) confirms the stretched shape of the complexes having a peak at short distances and an elongated tail^37^. The *R*_*g*_ of the complexes determined by Guinier analysis (Supplementary Fig. 4a) show differences between apo (*R*_*g*_ 3.3 nm) and cyclodextrin-bound (*R*_*g*_ 3.6 nm) samples indicating that β-cyclodextrin has an effect on the overall size of the complex. The maximal protein complexes dimensions, *D*_max_, were determined to be 11.9 nm and 12.3 nm in the binary and ternary complexes, respectively. Dimensionless Kratky plot representation of the scattering data enables to qualitatively compare SAXS profiles of objects with apparently different *R*_*g*_ values^38^. The Kratky plot representation of the scattering curves shows the presence of a characteristic parabola-like peak that confirms the compact structures, and a slight increase at higher *q* values, which indicates a certain flexibility of the in-solution structures. This suggests that PP1/PTG domains may not adopt a unique arrangement in solution (Fig. 5c).

We evaluated the experimental scattering curves of the PTG-PP1 complexes with the atomic structures obtained in the present study and found large discrepancies between the experimental and theoretical scattering curves (χ^2^ value larger than 50, Supplementary Fig. 4c) in the *q* 0.05 to 3 nm^−1^ region. The ternary complex shows a theoretical *R*_*g*_ value of 2.67 nm *in cristallo* compared to the experimental 3.6 nm in solution: this clearly confirms that different conformations coexist in solution. The observation that the PTG CBM has little to no affinity for PP1 suggests that this domain establishes transient interactions with PP1 and may explore different positions while linked to PP1 through the two PTG segments 83–102 (RVVF) and 111–128 (SALK). Given the evidence of this intrinsic flexibility within the PP1/PTG complexes, we used Ensemble Optimization Method (EOM)-based rigid body modeling to describe the different PTG CBM21 orientations in both apo and β-cyclodextrin conditions. EOM gives valuable information, such as *R*_*g*_ and *D*_max_ distributions, in the case of proteins with flexible domains. The EOM analysis was performed on SAXS curves in which the *q*-range region was fixed from 0.05 to 3 nm^−1^ and using the high-resolution crystal structures of PTG and PP1 domains obtained in this study. The PTG segments 83–102 and 111–128 were left bound to PP1, while no restraints were applied to both apo and β-cyclodextrin-bound CBM21. We found that these entry parameters yielded good quality fits as visible in the log I(*q*) versus *q* plots of the EOM curves overlaid with the experimental data and in the error-weighted residual difference plots (Fig. 5a). By comparison, the EOM modeling of the ternary complex with constraints applied to the CBM/PP1 interface as observed in the crystal structure gives significant discrepancy with experimental data (*χ*^2^ 22.2, Supplementary Fig. 4c). The EOM fitting of the ternary complex with unconstrained CBM/PP1 interface gives a *χ*^2^ value of 1.9 compared to a value of 1.0 observed for the binary complex in the same setting. Minor differences in the fitting *versus* experimental curve observed in the ternary complex are located in 0.08–0.12 nm^−1^ *q*-range region, as highlighted in Supplementary Fig. 4d. The higher *χ*^2^ for the ternary complex derives from the CBM21 domain interacting with β-cyclodextrin. The CBM21 bound to β-cyclodextrin at site II was used as rigid body in the EOM modeling. This does not take into consideration the binding equilibrium and the local conformational changes at site II (*K*_D_ in the double-digit micromolar range with fast *k*_on_ and *k*_off_, as determined by ITC and GCI) and the possible binding at site I in our experimental conditions, which, as discussed above in relation to the ITC titration, cannot be excluded.

Size distributions (*R*_*g*_ and *D*_max_) of apo *versus* cyclodextrin-bound complexes provided a qualitative assessment of the structural features of the complexes through direct comparison of the multimodal distributions of the selected ensembles and the pools (Supplementary Fig. 5a, b). The pools are composed of 10,000 possible conformations in order to approximate the otherwise infinite conformational space adopted by proteins with flexible domains. Sub-ensembles of conformers coexisting in solution are then selected guided by the fit to the experimental SAXS data. The EOM tool finally produces feasible 3D models (e.g. avoiding steric clashes and maintaining chain connectivity) that cover the possible conformational space and incorporate high-resolution information from complementary techniques, such as X-ray protein crystallography^39^.

The ensemble distributions predict multiple orientations adopted by the CBM both in the apo and in the cyclodextrin-bound form. In the binary complex the analysis predicted *R*_*g*_ and *D*_max_ values of 3.41 ± 0.27 nm and 13.3 ± 1.93 nm, respectively; while in the ternary complex of 3.57 ± 0.36 nm and 14.49 ± 2.6 nm, respectively. Notably, these structural parameters are in good agreement with *R*_*g*_ and *D*_max_ values obtained from Guinier and P(*r*) analysis.

An illustrative structure of the ternary complex generated by EOM is displayed in Fig. 5d, showing a CBM21-protruding PTG conformation. These data support the hypothesis that in solution the CBM explores several orientations variously located around the PP1-binding site observed *in cristallo* (Supplementary Fig 5c, d). Accordingly, the overall ab initio shapes of the protein complexes confirm the observation that PTG-PP1 has an extended structure and an envelope that fits the EOM models obtained in this study (Supplementary Fig. 5e, f).

### AlphaFold correctly predicted the structure of PTG CBM21 but not the organization of the N-terminal PP1-interacting region

With the groundbreaking development of AlphaFold and the release of the corresponding prediction database^40,41^, comparisons between newly determined structures and the released models would help refine and further develop the current methodology. Here we notice that AlphaFold excellently predicted the structure of the PTG CBM21 with an average RMSD over Cα atoms of 0.61 Å. Interestingly, the β3-β4 and the β6-β7 loops are correctly predicted in the cyclodextrin-bound conformation with Phe80 exposed to the solvent almost in the exact location when it becomes buried by the β-cyclodextrin molecule. The N-terminal region outside the CBM21 configures as an intrinsically disordered region (IDR) folding on the partner protein. The prediction significantly diverges from the PP1-bound conformation (Supplementary Fig. 6); this is partially reflected in the confidence score, dropping from the high/very high assigned to the CBM21 to the low/very low of the 102–132 region (including the SALK sequence predicted in helical conformation) and the medium/high of the 83–101 segment containing the PP1-binding elements βA (with the RVXF signature), ηA and βB.

We emphasize that this is perfectly in line with what reported by the AlphaFold team about the much lower accuracy for regions in which the chain has a high percentage of heterotypic, cross-chain contacts^40^. AlphaFold is expected to become applicable to predicting full hetero-complexes in a future system and that this will remove the difficulty with protein chains that have a large number of hetero-contacts^41^. The PP1/PTG structure presented here could be part of the future training set.

## Discussion

The data presented above allow drawing many conclusions starting to unveil, at the molecular level, the mechanical and regulatory aspects concerning the PTG/PP1 complex.

The crystal structure of the PTG CBM exhibits a typical immunoglobulin-like fold, with few extra secondary structure elements, being more similar to GL than to GM. It has a leading carbohydrate-binding site (site II) with a putative ancillary interaction site that could participate in binding of long-chain polysaccharides in analogy with *R. oryzae* CBM21. The interaction with carbohydrates is dominated by polar contacts with an essential enthalpic contribution balanced by an entropic penalty, fixing the affinity in the double-digit micromolar range.

The crystal structure of the PTG/PP1 complex reveals that PTG binding to PP1 is dictated by the N-terminal region outside the CBM21, dominated by the so-called RVXF and ΦΦ motifs that extend the PP1 β-sheet by organizing in two β-strands connected by a 3_10_ helix. The SALK sequence, peculiarly located C-terminal to the RVXL motif, further but minorly contributes to the binding affinity, while the CBM21 does not.

In the complex with PP1, the PTG CBM21 appears to fluctuate in solution, variously orienting itself with respect to PP1. This is confirmed by the SAXS data and the undetectable binding of the isolated CBM21 to PP1. Furthermore, the tetrameric assembly observed in the PP1/PTG crystal structure requires the SALK sequence to transiently detach from the PP1 surface. Whether this tetrameric structure is merely a crystallographic artifact or reflects a physiological assembly driven by the formation of larger complexes in glycogen granules remains to be established. Notably, almost all PTG/PP1 partners are multimeric, including laforin, GYS and PYG.

## Methods

### Protein expression and purification

#### PTG CBM21

The DNA sequence containing CBM21 (residues 132–264) was inserted by LIC cloning into the pNIC28-Bsa4 vector with N-terminal 6x His-tag and TEV cleavage site. The plasmid was transformed into BL21 (DE3) competent cells and the protein was expressed in autoinduction medium ZYM-5052 at 18 °C for 18–20 h. Cell culture was harvested by centrifugation at 3000 × *g* at 4 °C and washed with cold 1xPBS + 10% glycerol. The pellet was resuspended in binding buffer (50 mM Tris pH 8, 0.5 M NaCl, 10% glycerol, 10 mM imidazole, 1x complete protease inhibitor cocktail EDTA-free (Roche), 10 mM MgCl_2_, 10 μg/ml DNaseI) and lysed by 2 cycles of homogenization at ~1000 bar (PandaPLUS 2000, GEA). The soluble fraction was separated by centrifugation at 30,000 × *g* at 4 °C for 1 h. The supernatant was incubated with Ni-NTA Agarose resin (Qiagen) for 1 h at 4 °C, the flow-through was collected by gravity, the resin was washed extensively with wash buffer (50 mM Tris pH 8, 0.5 M NaCl, 10% glycerol, 10 mM imidazole) and the protein eluted with the same buffer containing 300 mM imidazole. Eluted protein was dialyzed O/N at 4 °C against 25 mM Hepes pH 7.4, 0.2 M NaCl, 1 mM DTT in the presence of TEV protease to remove the 6xHis-tag. The cleaved protein was further purified by negative affinity using Ni-NTA Agarose resin and loaded on anion exchange column 5 ml HiTrap Q HP (Cytiva). FT containing the protein was concentrated on a 10k MWCO centrifugal concentrator (PierceTM, ThermoScientific) and loaded on size-exclusion chromatography column Superdex 75 16/60 in 25 mM Hepes pH 7.4, 0.2 M NaCl, 5% glycerol, 1 mM DTT. Fractions containing PTG CBM21 were pooled together, flash frozen and stored at −80 °C.

#### PTG^70-264^

The *E. coli* codon-optimized gene encoding for human PTG (residues 70–264, purchased from GenScript) was cloned into pGEX-6-3 vector using BamHI and NotI. The construct contains an N-terminal GST tag cleavable by 3 C protease. The protein was co-expressed together with the GroEL/ES chaperonins (pGro7 vector, Takara Bio). PTG^70-264^ was produced in LB medium supplemented with antibiotics and 0.5 mg/ml of arabinose, following the induction at OD_600_~ 0.6 with 0.1 mM 1-thio-β-D-galactopyranoside (IPTG) and expression at 18 °C for 18–20 h. Cell pellet was harvested as described above, resuspended in lysis buffer (50 mM Tris pH 8, 0.5 M NaCl, 10% glycerol, 10 mM DTT, 10 mM MgCl_2_, 10 µg/ml DNase I, 1x complete protease inhibitor cocktail). Cells were lysed and soluble fraction recovered as previously described. The supernatant was incubated with Protino Glutathione Agarose 4B resin (MACHEREY-NAGEL) for 1 h at 4 °C in mild agitation. The resin was loaded on polypropylene column (Bio-Rad) and extensively washed with wash buffer 1 (50 mM Tris pH 8, 0.5 M NaCl, 10% glycerol, 2 mM DTT), followed by 2-3 column washes with buffer 2 (100 mM Tris pH 7.5, 50 mM sucrose, 10 mM KCl, 25% glycerol, 20 mM MgCl_2_, 5 mM ATP, 1 mM DTT) to remove the chaperonins excess. The resin was rinsed with additional 10 column volumes (CV) of buffer 1. GST-tag was removed on-column by adding 3 C protease directly to the resin in 1 CV of wash buffer 1 and incubated overnight at 4 °C. Flow-through fraction containing cleaved PTG was collected and concentrated using a 10k MWCO centrifugal concentrator and further purified on size-exclusion chromatography column Superdex 200 10/300 GL (Cytiva). The PTG fractions were pooled together, concentrated using a 10k MWCO concentrator, flash-frozen in liquid nitrogen and stored at −80 °C.

*PP1*^*7-330*^ vector with N-terminal 6xHis-tag and TEV cleavage site was a gift from Wolfgang Peti (Addgene plasmid # 26566). A stop codon was introduced by site-directed mutagenesis to obtain the construct 7–300. The protein was expressed in BL21 (DE3) pGro7 cells (described above) in LB supplemented with antibiotics, 0.5 mg/ml of arabinose and 1 mM MnCl_2_. Induction and cell harvest were performed as described for PTG 70–264. Cell pellet was resuspended in lysis buffer (50 mM Tris pH8, 0.5 M NaCl, 10% glycerol, 1 mM MnCl_2_, 10 mM MgCl_2_, 10 µg/ml DNase I, 1x complete protease inhibitor cocktail). Following lysis and removal of the insoluble fraction, the supernatant was incubated with Ni-NTA Agarose resin for 1 h at 4 °C. The flow-through was collected by gravity, the resin was washed extensively with wash buffer (50 mM Tris pH 8, 0.5 M NaCl, 10% glycerol, 1 mM MnCl_2_, 10 mM imidazole) and the protein was eluted with the same buffer containing 300 mM imidazole. His-tag was removed by TEV protease O/N at 4 °C in dialysis against the buffer 50 mM Tris pH 8, 0.5 M NaCl, 10% glycerol, 1 mM MnCl_2_. The next day precipitation was observed and removed by centrifugation at 30,000 × *g*, 20 min at 4 °C. The protein was later loaded on SEC column Superdex 200 26/60 (Cytiva) equilibrated with dialysis buffer. Fractions containing PP1^7-300^ were pooled together and flash frozen. Construct PP1^7-330^ was produced following the same protocol.

#### PTG^70-264^/PP1 complex

For complex production, BL21 (DE3) pGro7 cells containing PP1^7-300^ were rendered competent and subsequently transformed with PTG 70–264. Expression was performed as described for PP1 7–300. Cell pellet was resuspended in buffer 50 mM Tris pH 8, 0.5 M NaCl, 10% glycerol, 1 mM MnCl_2,_ 0.5 mM TCEP, 10 mM MgCl_2_, 10 µg/ml DNase I, 1x complete protease inhibitor cocktail, lysed and clarified. Purification was performed as described for PTG 70–264 with buffers supplemented with 1 mM MnCl_2_ and 0.5 mM TCEP instead of DTT. The tags were removed on-column by adding 3 C protease and TEV protease directly to the resin in 1 CV of wash buffer 1 and incubated overnight at 4 °C. Flow-through (FT) fraction containing cleaved PTG/PP1 complex was further incubated with Ni-NTA Agarose resin to remove His-tag and uncleaved PP1 7–300. FT was collected and concentrated using a 10k MWCO centrifugal concentrator and further purified on size-exclusion chromatography column Superdex 200 10/300 GL. The PTG/PP1 protein complex fractions were pooled together and flash-frozen in liquid nitrogen and stored at −80 °C.

For SAXS analysis, PTG^70-264^/PP1^7-330^ complex was produced as described above without His-tag removal and Ni-NTA negative affinity steps.

### Crystallization, diffraction data collection, and structure solution

#### PTG CBM21

The protein was incubated with a 3x molar excess of β-cyclodextrin (Calbiochem) and concentrated to 50 mg/ml. Crystals grew in sitting drops in two different conditions: 3 M NaCl, 0.1 M BIS-Tris pH 5.5 and 0.1 M MMT buffer pH 4, 25% PEG 1500 at 20 °C. The mother liquor solution supplemented with 20% glycerol was used for cryo-protection.

#### PP1^7-300^/PTG^81-107^

PTG^81-107^ peptide was purchased from ProteoGenix SAS and resuspended in 20 mm Tris pH 8, 25 mM NaCl. PP1^7-300^ was diluted 10x with buffer 25 mM Tris pH 8, 50 mM NaCl, 1 mM MnCl_2_, 0.5 mM TCEP and incubated together with PTG^81-107^ peptide with 1.1 molar excess for 1 h at 4 °C. The protein/peptide complex was subsequently concentrated at 8 mg/ml. Crystals grew in sitting drop in 0.1 M Tris pH 8, 1.6 M lithium sulfate, 1.4 M sodium malonate pH 6.0 at 20 °C.

Electron densities (F_o_-F_c_ polder OMIT map) for β-cyclodextrin, RVVF peptide, SALK peptide and dimerization strands are shown in Supplementary Fig. 7.

#### PP1^7-300^/PTG^70-264^

β-cyclodextrin powder was directly dissolved in the PP1^7-300^/PTG^70-264^ protein complex solution at a final concentration of 5 mM and the whole complex was further concentrated up to 10 mg/ml. Small, badly diffracting crystals were obtained in condition 0.1 M Na citrate pH 6, 2 M NaCl and used for seeding experiments. Big crystals grew in sitting drop in 1.0 M sodium malonate pH 5.0, 0.1 M sodium acetate trihydrate pH 4.5 at 4 °C, however still diffracting poorly (5–10 Å). The crystal quality improved after dehydration O/N in solution with 10% higher precipitant concentration supplemented with 10% glycerol and 1 mM β-cyclodextrin. The crystals were cryo-protected in the dehydrating solution with 20% glycerol or xylitol and flash-frozen in liquid nitrogen.

All diffraction data were collected on XRD2 beamline at Elettra Sincrotrone Trieste (Italy), integrated and scaled with XDS^42^ (as part of the Global Phasing autoPROC pipeline 1.0.5) and merged with Aimless^43^ (CCP4 suite).

The PTG-CBM21 structure was solved by molecular replacement with Phaser^44^ (CCP4 suite) using the GL structure 2EEF as search model, following the removal of the longest loops. Cycles of manual rebuilding with Coot^45^ and refinement with Phenix^46^ allowed filling all gaps and reconstituting the correct sequence, while the evident residual density was used to include the cyclodextrin molecule.

The *PP1*^*7-300*^*/PTG*^*81-107*^ structure was solved by molecular replacement with Phaser using the PP1 structure 6ZEG as search model. The PTG peptide was manually built taking advantage of the similar GM peptide in the 6DNO structure. Refinement was conducted with Phenix.

The *PP1*^*7-300*^*/PTG*^*70-264*^ structure was solved by molecular replacement with Phaser using the two above structures as search models. Data were anisotropically corrected with Staraniso (http://staraniso.globalphasing.org/cgi-bin/staraniso.cgi). Clear positive electron density was used to build the missing PTG residues. Cycles of manual and automatic refinement were performed by using Coot and Phenix, respectively.

Data collection and structure refinement statistics are reported in Supplementary Table 3.

### Small-angle X-ray scattering data collection and analysis

The experiments were performed at the ESRF BioSAXS beamline BM29, Grenoble, France^47^. The PTG-PP1 and PTG-PP1 + β-cyclodextrin complexes were measured by SEC-SAXS; a volume of 50 μL of protein per sample at 4 mg/ml (without β-cyclodextrin) and 6.5 mg/ml (with β-cyclodextrin) was loaded on an AdvanceBio SEC 130 Å (4.6 × 50 mm) column (Agilent) via a high-performance liquid chromatography device (HPLC, Shimadzu) attached directly to the sample-inlet valve of the BM29 sample changer^35^. All the samples were measured in buffer 50 mM Tris pH 8, 0.5 M NaCl, 10% glycerol, 1 mM DTT (with or without β-cyclodextrin at the final concentration of 5 mM) at 20 °C. The column was equilibrated with 3 CV to obtain a stable background signal before measurement. A flow rate of 0.3 ml/min was used for all sample measurements. All SAXS data were collected at a wavelength of 0.99 Å using a sample-to-detector (PILATUS 2 M; DECTRIS) distance of 2.867 m. The scattering of pure water was used to calibrate the intensity to absolute units^48^. All parameters for SAXS analysis are described in Supplementary Table 4, according to SAXS community guidelines^49^. Briefly, data reduction was performed automatically using the EDNA pipeline^50^. In the SEC-SAXS chromatograms, frames in regions of stable *R*_*g*_ were selected with CHROMIXS and averaged using PRIMUS to yield a single averaged frame per protein sample. Analysis of the overall parameters was carried out by PRIMUS from ATSAS 3.0.4 package^29^. For PTG-PP1 and PTG-PP1 + β-cyclodextrin complexes, the pair distance distribution functions, *P*(r), were used to calculate ab initio models in P1 symmetry with DAMMIF^51^ (ATSAS package). For rigid bodies structural modeling, ensemble optimization method (EOM, ATSAS package) analysis^48–52^ was conducted using the X-ray crystal structure of PTG-PP1 + β-cyclodextrin complex obtained in the present study. During the EOM analysis, the PTG segments 83–102 and 111–128 were left bound to PP1 (residues 7–299), as observed in the crystal structure. The PTG domain from residue 133 to 259 was left unbound to PP1, modeled as a flexible domain linked to the rest of the PTG protein *via* the linker from residue 128–132). The rest of the aminoacidic sequences were modeled as flexible segments by EOM: PP1 300–330, PTG 70–82, 103–110 and 128–132. The missing connections linking the rigid bodies and the EOM-modeled segments were added using MODELLER, a tool implemented in UCSF CHIMERA software^53,54^. The quality of EOM fits over the *q*-range of experimental SAXS curves was assessed with CORMAP analysis from ATSAS 3.0.4 package and with error-weighted residual difference plots of [*I*_exp_(*q*) - *I*_comp_(*q*)]/*σ*_exp_(*q*), corresponding to the difference between the experimental and computed intensities weighted by the experimental uncertainty^49^. Five independent EOM internal replicas were carried out per each apo and β-cyclodextrin-bound PTG-PP1 SAXS curves and the distributions of selected *R*_*g*_ and *D*_max_ ensembles were averaged. Plots and protein models were generated using OriginPro 9.0 and UCSF Chimera software, respectively.

### Grating-coupled Interferometry

Grating-coupled interferometry (GCI) experiments were performed using the Creoptix WAVE system, a new generation instrument for the high-sensitivity kinetics and affinity analysis of label-free molecular interactions based on the waveguide interferometry technology. Waveguide interferometry exceeds the sensitivity levels of Surface Plasmon Resonance (SPR). In contrast to SPR, GCI provides an evanescent field that penetrates less into the bulk and extends the light-to-sample interaction length for superior signal-to-noise ratios^55^. GCI allows getting high resolution even at very low signal levels with low noise without artificial data averaging and reliable kinetics below 1 pg/mm^2^. By the lower immobilization levels that are required, it avoids mass transport limitations. It covers the broadest kinetic range with the capability of analyzing of the ultra-fast transition times (i.e. 150 ms). It allows the reliable determination of off-rates of 5 s^–1^ and even faster, enabling off-rate screening of weakly binding analytes. GCI can be applied to a wide range of pharmacological targets, with improved results on very low molecular weight compounds, to the dimensions of metal ions^56^.

WAVE chips are composed of four channels or cells. Borate buffer (100 mM sodium borate pH 9.0, 1 M NaCl) was used for chip conditioning. To evaluate the kinetics of the PTG interaction with β-cyclodextrin and PP1, PTG^70-264^ (20 µg/ml in buffer acetate pH 5.0) was immobilized on 4PCH WAVE chips (quasiplanar polycarboxylate surface; Creoptix) by standard amine coupling according to the manufacturer’s instructions at a density of 1800 pg/mm^2^ and 1000 pg/mm^2^ for β-cyclodextrin and PP1^7-330^, respectively (Supplementary Figs. 8, 9). Regeneration-free injections of a 1:3 dilution series from 500 µM for β-cyclodextrin in running buffer (10 mM Hepes pH 7.2, 150 mM NaCl, 0.005% Tween 20) were performed at 25 °C, using a flow rate of 100 μl/min. Similarly, regeneration-free injections of a 1:2 dilution series from 400 nM for PP1^7-330^ in the same running buffer were performed at 25 °C, using a flow rate of 30 μl/min. Experiments to evaluate the kinetics of the interaction between PP1^7-330^ and the two PTG-derived peptides (PTG^81-107^ and PTG^81-132^, ProteoGenix SAS) were performed reversibly capturing 6xHis-tagged PP1^7-330^ (5 μg/ml in running buffer 10 mM Hepes pH 7.2, 150 mM NaCl, 0.005% Tween 20, 0.2 mg/ml BSA) on 4PCP-NTA WAVE chips according to the manufacturer’s instructions at a density of 3600 pg/mm^2^ and 1600 pg/mm^2^ for peptide PTG^81-107^ and PTG^81-132^, respectively (Supplementary Figs. 10, 11). Regeneration-free injections of a 1:2 dilution series from 300 nM for the two peptides in running buffer were performed at 25 °C, using a flow rate of 100 μl/min. Blank injections were used for double referencing and DMSO calibration curve for bulk correction. Analysis and correction of the obtained data were performed using the Creoptix WAVE control software (correction applied: X and Y offset; DMSO calibration; double referencing). Mass transport binding models with bulk correction were used to fit all experiments. All the experiments have been performed at least in triplicate.

### Isothermal titration calorimetry (ITC)

ITC experiments were performed using an ITC 200 microcalorimeter (Malvern Panalytical, UK) at 25 °C with a stirring speed of 750 rpm in 25 mM Hepes pH 7.4, 0.2 M NaCl, 5% glycerol with 180 s separating the injections. β-cyclodextrin was dissolved in the running buffer and two datasets were collected, one with PTG CBM21 concentration at 100 μM and β-cyclodextrin at 3 mM with 40 injections of 1 µl, the other at 200 μM and 4 mM, respectively, with 80 injections of 1 µl. A blank run with only the buffer was carried out. Data analysis was done with ORIGIN subtracting the blank titrations fitting ‘one site’ and ‘two sites’ models.

### Reporting summary

Further information on research design is available in the Nature Research Reporting Summary linked to this article.

## Supplementary information

Supplementary Information

Reporting Summary

## Data Availability

Data and structures generated in this study have been deposited in the PDB database under accession codes: 7QF7 (PTG-CBM21 in complex with β-cyclodextrin, orthorhombic crystal form), 7QFA (PTG-CBM21 in complex with β-cyclodextrin, monoclinic crystal form), 7QFB (PP1/PTG^81-107^) and 7QM2 (PP1/PTG^70-264^). SAXS data have been deposited in the Small Angle Scattering Biological Data Bank (SASBDB) under accession numbers SASDNF2 and SASDNG2 for PTG-PP1 and PTG-PP1+β-cyclodextrin, respectively.

## References

[1] Roach PJ, Depaoli-Roach AA, Hurley TD, Tagliabracci VS (2012). Glycogen and its metabolism: some new developments and old themes. Biochem. J..

[2] GTEx Consortium. (2020). The GTEx Consortium atlas of genetic regulatory effects across human tissues. Science.

[3] Bak LK, Walls AB, Schousboe A, Waagepetersen HS (2018). Astrocytic glycogen metabolism in the healthy and diseased brain. J. Biol. Chem..

[4] Vilchez D (2007). Mechanism suppressing glycogen synthesis in neurons and its demise in progressive myoclonus epilepsy. Nat. Neurosci..

[5] Gentry MS, Worby CA, Dixon JE (2005). Insights into Lafora disease: malin is an E3 ubiquitin ligase that ubiquitinates and promotes the degradation of laforin. Proc. Natl Acad. Sci. USA.

[6] Minassian BA (1998). Mutations in a gene encoding a novel protein tyrosine phosphatase cause progressive myoclonus epilepsy. Nat. Genet..

[7] Chan EM (2003). Mutations in NHLRC1 cause progressive myoclonus epilepsy. Nat. Genet..

[8] Nitschke F, Ahonen SJ, Nitschke S, Mitra S, Minassian BA (2018). Lafora disease—from pathogenesis to treatment strategies. Nat. Rev. Neurol..

[9] Verhalen B, Arnold S, Minassian BA (2018). Lafora disease: a review of molecular mechanisms and pathology. Neuropediatrics.

[10] Turnbull J (2011). PTG depletion removes Lafora bodies and rescues the fatal epilepsy of Lafora disease. PLoS Genet..

[11] Turnbull J (2014). PTG protein depletion rescues malin-deficient Lafora disease in mouse. Ann. Neurol..

[12] Kumar GS (2018). Identification of the substrate recruitment mechanism of the muscle glycogen protein phosphatase 1 holoenzyme. Sci. Adv..

[13] Tung JY (2008). Crystal structures of the starch-binding domain from Rhizopus oryzaeglucoamylase reveal a polysaccharide-binding path. Biochem. J..

[14] Chu CH (2014). Crystal structures of starch binding domain from Rhizopus oryzaeglucoamylase in complex with isomaltooligosaccharide: insights into polysaccharide binding mechanism of CBM21 family. Proteins.

[15] Chou WI, Pai TW, Liu SH, Hsiung BK, Chang MD (2006). The family 21 carbohydrate-binding module of glucoamylase from Rhizopusoryzae consists of two sites playing distinct roles in ligand binding. Biochem. J..

[16] Jiang TY (2012). Two unique ligand-binding clamps of Rhizopus oryzae starch binding domain for helical structure disruption of amylose. PLoS ONE.

[17] Yu J, Deng T, Xiang S (2018). Structural basis for protein phosphatase 1 recruitment by glycogen-targeting subunits. FEBS J..

[18] Walker KS, Watt PW, Cohen P (2000). Phosphorylation of the skeletal muscle glycogen-targetting subunit of protein phosphatase 1 in response to adrenaline in vivo. FEBS Lett..

[19] Yamamoto-Honda R (2000). Overexpression of the glycogen targeting (G(M)) subunit of protein phosphatase-1. Biochem. Biophys. Res. Commun..

[20] Liu J, Wu J, Oliver C, Shenolikar S, Brautigan DL (2000). Mutations of the serine phosphorylated in the protein phosphatase-1-binding motif in the skeletal muscle glycogen-targeting subunit. Biochem. J..

[21] Dent P, Campbell DG, Hubbard MJ, Cohen P (1989). Multisite phosphorylation of the glycogen-binding subunit of protein phosphatase-1G by cyclic AMP-dependent protein kinase and glycogen synthase kinase-3. FEBS Lett..

[22] Gasa R (2000). Distinctive regulatory and metabolic properties of glycogen-targeting subunits of protein phosphatase-1 (PTG, GL, GM/RGl) expressed in hepatocytes. J. Biol. Chem..

[23] Akimov V (2018). UbiSite approach for comprehensive mapping of lysine and N-terminal ubiquitination sites. Nat. Struct. Mol. Biol..

[24] Ragusa MJ (2010). Spinophilin directs protein phosphatase 1 specificity by blocking substrate binding sites. Nat. Struct. Mol. Biol..

[25] Fedoryshchak RO (2020). Molecular basis for substrate specificity of the Phactr1/PP1 phosphatase holoenzyme. Elife.

[26] Chen R (2015). G-actin provides substrate-specificity to eukaryotic initiation factor 2α holophosphatases. Elife.

[27] Choy MS (2014). Understanding the antagonism of retinoblastoma protein dephosphorylation by PNUTS provides insights into the PP1 regulatory code. Proc. Natl Acad. Sci. USA.

[28] Bajaj R, Bollen M, Peti W, Page R (2018). KNL1 binding to PP1 and microtubules is mutually exclusive. Structure.

[29] Hurley TD (2007). Structural basis for regulation of protein phosphatase 1 by inhibitor-2. J. Biol. Chem..

[30] Roy J, Cyert MS (2009). Cracking the phosphatase code: docking interactions determine substrate specificity. Sci. Signal..

[31] Bollen M, Peti W, Ragusa MJ, Beullens M (2010). The extended PP1 toolkit: designed to create specificity. Trends Biochem. Sci..

[32] Hendrickx A (2009). Docking motif-guided mapping of the interactome of protein phosphatase-1. Chem. Biol..

[33] Heroes E (2013). The PP1 binding code: a molecular-lego strategy that governs specificity. FEBS J..

[34] Liu D (2010). Regulated targeting of protein phosphatase 1 to the outer kinetochore by KNL1 opposes Aurora B kinase. J. Cell. Biol..

[35] Manalastas-Cantos K (2021). ATSAS 3.0: expanded functionality and new tools for small-angle scattering data analysis. J. Appl. Crystallogr..

[36] Brennich ME, Round AR, Hutin S (2017). Online size-exclusion and ion-exchange chromatography on a SAXS beamline. J. Vis. Exp..

[37] Putnam CD, Hammel M, Hura GL, Tainer JA (2007). X-ray solution scattering (SAXS) combined with crystallography and computation: defining accurate macromolecular structures, conformations and assemblies in solution. Q. Rev. Biophys..

[38] Durand D (2010). NADPH oxidase activator p67(phox) behaves in solution as a multidomain protein with semi-flexible linkers. J. Struct. Biol..

[39] Tria G, Mertens HDT, Kachala M, Svergun DI (2015). Advanced ensemble modelling of flexible macromolecules using X-ray solution scattering. IUCrJ.

[40] Tunyasuvunakool K (2021). Highly accurate protein structure prediction for the human proteome. Nature.

[41] Jumper J (2021). Highly accurate protein structure prediction with AlphaFold. Nature.

[42] Kabsch W (2010). XDS. Acta Crystallogr. D: Biol. Crystallogr..

[43] Evans PR, Murshudov GN (2013). How good are my data and what is the resolution?. Acta Crystallogr. D: Biol. Crystallogr..

[44] McCoy AJ (2007). Phaser crystallographic software. J. Appl. Crystallogr..

[45] Emsley P, Lohkamp B, Scott WG, Cowtan K (2010). Features and development of Coot. Acta Crystallogr. D: Biol. Crystallogr..

[46] Adams PD (2010). PHENIX: a comprehensive Python-based system for macromolecular structure solution. Acta Crystallogr. D: Biol. Crystallogr..

[47] Pernot P (2013). Upgraded ESRF BM29 beamline for SAXS on macromolecules in solution. J. Synchrotron Radiat..

[48] Orthaber D, Bergmann A, Glatter O (2000). SAXS experiments on absolute scale with Kratky systems using water as a secondary standard. J. Appl. Crystallogr..

[49] Trewhella J (2017). 2017 publication guidelines for structural modelling of small-angle scattering data from biomolecules in solution: an update. Acta Crystallogr. D: Struct. Biol..

[50] Brennich ME (2016). Online data analysis at the ESRF bioSAXS beamline, BM29. J. Appl. Crystallogr..

[51] Franke D, Svergun DI (2009). DAMMIF, a program for rapid ab-initio shape determination in small-angle scattering. J. Appl. Crystallogr..

[52] Bernado P, Mylonas E, Petoukhov MV, Blackledge M, Svergun DI (2007). Structural characterization of flexible proteins using small-angle X-ray scattering. J. Am. Chem. Soc..

[53] Pettersen EF (2004). UCSF Chimera—a visualization system for exploratory research and analysis. J. Comput. Chem..

[54] Šali A, Blundell TL (1993). Comparative protein modelling by satisfaction of spatial restraints. J. Mol. Biol..

[55] Patko D, Cottier K, Hamori A, Horvath R (2012). Single beam grating coupled interferometry: high resolution miniaturized label-free sensor for plate based parallel screening. Opt. Express.

[56] Jankovics H (2020). Grating-coupled interferometry reveals binding kinetics and affinities of Ni ions to genetically engineered protein layers. Sci. Rep..

[57] Svergun DI, Pedersen JS (1994). Propagating errors in small-angle scattering data treatment. J. Appl. Crystallogr..

[58] Larsen AH, Pedersen MC (2021). Experimental noise in small-angle scattering can be assessed using the Bayesian indirect Fourier transformation. J. Appl. Crystallogr..

