# Supplementary material for: Molecular architecture of the glycogen- committed PP1/PTG holoenzyme
Source: Nat Commun. 2022 Oct 19;13:6199. doi: 10.1038/s41467-022-33693-z (PMC9582199; doi:10.1038/s41467-022-33693-z)
Supplement: Supplementary file 1 — Supplementary Information [file 41467_2022_33693_MOESM1_ESM.pdf]

SUPPLEMENTARY INFORMATION

Molecular architecture of the glycogen-committed PP1/PTG holoenzyme

Marta Stefania Semrau[1,2], Gabriele Giachin[3], Sonia Covaceuszach[4], Alberto Cassetta[4], Nicola Demitri[5], Paola Storici[2] and Graziano Lolli[1]

[1]Department of Cellular, Computational and Integrative Biology - CIBio, University of Trento, via Sommarive 9, 38123 Povo - Trento, Italy

[2]Protein Facility, Elettra Sincrotrone Trieste S.C.p.A, SS 14 - km 163,5 in AREA Science Park, 34149 Basovizza - Trieste, Italy

[3]Department of Chemical Sciences (DiSC), University of Padua, via F. Marzolo 1, 35131 Padova, Italy

[4]Institute of Crystallography - C.N.R.- Trieste Outstation. Area Science Park – Basovizza, S.S.14 - Km. 163.5, I-34149 Trieste, Italy

[5]XRD2 Beamline, Elettra Sincrotrone Trieste S.C.p.A, SS 14 - km 163,5 in AREA Science Park, 34149 Basovizza - Trieste, Italy

Correspondence to Paola Storici and Graziano Lolli: paola.storici@elettra.eu; graziano.lolli@unitn.it.

Supplementary Table 1. Thermodynamic parameters determined by ITC for the interaction of β-cyclodextrin with PTG. The values are the average of two separate runs with errors shown. Superscript 1 and 2 refer to the different sites of the 'two sites' models. Thermodynamic parameters for the first site are consistent independently of the binding model. ITC curve-fitting performed with the two-site model returns inconsistent stoichiometry for the second site and larger error values. Fixing the stoichiometry in the two sites model allows the parametrization of a second site; given the poor binding constant, this site is either spurious or configures as an ancillary site which binding to complex polysaccharides is cooperatively dictated by the primary interaction at site one.

Model	N^1	K_D^1 (μM)	ΔH^1 (kcal mol^{-1})	ΔS^1 (cal mol^{-1} K^{-1})	N^2	K_D^2 (μM)	ΔH^2 (kcal mol^{-1})	ΔS^2 (cal mol^{-1} K^{-1})	χ^2 *10^3
1 Site	1.09 ± 0.03	59 ± 5	-21.7 ± 0.9	-53					4.415 11.34
2 Sites	1.06 ± 0.05	63 ± 6	-18 ± 4	-40	0.3 ± 0.1	3 ± 2	-16 ± 2	-29	1.841 6.180
2 Sites fixed	1.0	49 ± 4	-24.6 ± 0.7	-62	1.0	346 ± 154	1.7 ± 0.8	21	2.872 9.714

χ^2 values are reported for the two experiments. The use of χ^2 in ITC data analysis when comparing different binding models is misleading. Indeed, χ^2 can be artificially lowered following an overparametrization, and then an overfitting, of the binding model. The high absolute value of χ^2 are due to the mathematical relationship used by the ORIGIN software in ITC data fitting. Indeed, χ^2 value of the fit is equal to the sum of the squares of the deviations of the theoretical curve from the experimental points divided by the number of degrees of freedom, without any statistical weight applied. The absolute value of the χ^2 therefore depends on the absolute scale of the data and it is quite large for interactions with a relevant enthalpic contribution, like the PTG/cyclodextrin case (for a complete description see: Malvern, MicroCal iTC200 system User Manual, MAN0560-01-EN-00 August 2014 version; paragraph 6.7, pag. 248). As reported in various worked examples described in the manual, χ^2 in the range $10^3 – 10^4$ are quite common for well-behaving experiments with large enthalpic contributions.

Supplementary Table 2. Kinetic parameters determined by GCI for the interaction of PTG with β-cyclodextrin and of PP1 with PTG or PTG peptides. The values are the average of three separate runs with errors shown.

Ligand	Analyte	k_{on} ($M^{-1}s^{-1}$)	k_{off} (s^{-1})	K_D (M)	χ^2
$PTG^{132-264\ (CBM)}$	β-cyclodextrin	$4.20 \pm 0.60 \times 10^{1}$	$1.55 \pm 0.05 \times 10^{-3}$	$37.3 \pm 7.0 \times 10^{-6}$	0.46±0.06
PTG^{70-264}	PP1	$5.00 \pm 0.30 \times 10^{4}$	$4.30 \pm 0.01 \times 10^{-3}$	$87.0 \pm 4.1 \times 10^{-9}$	0.73±0.07
PP1	PTG^{81-107}	$2.08 \pm 0.19 \times 10^{5}$	$2.95 \pm 0.77 \times 10^{-3}$	$14.1 \pm 2.4 \times 10^{-9}$	0.51±0.05
PP1	PTG^{81-132}	$3.57 \pm 0.78 \times 10^{5}$	$2.60 \pm 0.66 \times 10^{-3}$	$7.7 \pm 2.0 \times 10^{-9}$	0.66±0.02

Supplementary Table 3. Crystallographic Data Collection and Refinement Statistics

	PTG-CBM21 orthorhombic	PTG-CBM21 monoclinic	PP1/PTG$^{81-107}$	PP1/PTG$^{70-264}$
Data Collection				
Space group	$P22_12_1$	I2	$P2_12_12_1$	$P6_122$
Unit-cell parameters (Å, °)	a = 38.59<br>b = 56.44<br>c = 70.55	a = 66.83<br>b = 42.87<br>c = 168.99<br>β = 100.67	a = 66.38<br>b = 68.34<br>c = 119.78	a = 153.60<br>b = 153.60<br>c = 285.62
Wavelength (Å)	0.9718	0.9718	0.9999	1.0000
Resolution (Å)	44.07-1.47 (1.50-1.47)	46.81-2.00 (2.05-2.00)	68.34-2.05 (2.11-2.05)	120.58-2.69 (2.88-2.69)
R_{merge} (%)	4.7 (98.1)	10.6 (95.3)	19.5 (152.2)	36.3 (324.7)
R_{meas} (%)	5.2 (105.8)	11.5 (103.5)	20.2 (157.9)	37.4 (334.4)
R_{pim} (%)	2.0 (39.1)	4.5 (39.9)	5.3 (41.5)	9.0 (79.8)
$<I/\sigma(I)>$	17.1 (2.1)	12.0 (2.2)	10.0 (2.1)	13.3 (1.5)
$CC^{1/2}$	0.999 (0.837)	0.998 (0.778)	0.997 (0.737)	0.997 (0.422)
Completeness (%)	99.9 (100.0)	99.0 (98.7)	100.0 (100.0)	Sph. 66.3 (18.9)<br>Ell. 95.2 (73.1)
Multiplicity	6.8 (7.1)	6.6 (6.6)	14.3 (14.3)	32.0 (33.9)
Refinement				
Resolution (Å)	44.07-1.47	41.51-2.00	59.92-2.05	52.30-2.69
R_{work}/R_{free} (%)	17.6/19.5	17.3/21.2	15.9/19.1	17.7-21.3
R.m.s. deviations				
Bond lengths (Å)	0.005	0.007	0.006	0.010
Bond angles (°)	0.94	1.06	0.92	1.23
PDB entry	7QF7	7QFA	7QFB	7QM2

Supplementary Table 4. SAXS results for the protein complexes investigated in this study

a) Sample details

Sample name	PTG-PP1	PTG-PP1 + β-cyclodextrin
Organism	*Homo sapiens sapiens*	
UniProt sequence ID (residues in construct)	PP1 (Serine/threonine-protein phosphatase PP1-alpha catalytic subunit): UniProt ID P62136 (7-330). PTG (Protein phosphatase 1 regulatory subunit 3C): UniProt ID Q9UQK1 (70-262)	
Ligand - PubChem CID	-	β-cyclodextrin (DEX) - 444041
Calculated molecular weight (Da)	36834.35 (PP1) + 23012.78 (PTG) = 59847.13	36834.35 (PP1) + 23012.78 (PTG) + 1135 (DEX) = 60982.13
Total frames (frames used for data analysis)	990 (577-611)	750 (490-548)
SEC column	AdvanceBio SEC 130 Å (4.6 x 50 mm) column (Agilent)	
Injected volume (μL)	50	
Loading concentration (mg/mL)	4	6.5
Flow rates (mL/min)	0.3	
SEC buffer	50 mM Tris pH 8, 0.5 M NaCl, 10% glycerol, 1 mM DTT	50 mM Tris pH 8, 0.5 M NaCl, 10% glycerol, 1 mM DTT, 5 mM β-cyclodextrin

b) SAXS data collection parameters

Instrument	ESRF BM29
Wavelength (Å)	0.99
q-range (Å)	0.004-0.5
Sample-to-detector distance (m)	2.867
Exposure time	2 sec/frame
Temperature (° C)	20
Detector	Pilatus3 X 2M (Dectris)
Flux (photons/s)	2×10^{12}
Beam size (μm)	100 x 100
Sample configuration	1.8 mm quartz glass capillary
Absolute scaling method	Comparison to water in sample capillary
Normalization	To transmitted intensity by beam-stop counter

(c) Structural parameters

	PTG-PP1	PTG-PP1 + β-cyclodextrin
**Guinier analysis**		
- I(0) (cm^{-1})	0.0143 ± 5.6E-05	0.11 ± 3.76E-04
- R_g (nm)	3.3 ± 0.02	3.57 ± 0.02
- *q*-range (nm^{-1}), point range	0.0198 – 01595, 11-64	0.0304 – 0.121, 14-47
***P*(r) analysis**		
- I(0) (cm^{-1})	0.0144	0.1107
- R_g (nm)	3.4	3.6
- Dmax (nm)	11.9	12.3
- *q*-range (nm^{-1}), point range	0.0139 – 3.36, 7-640	0.006 – 2.71, 14-510
- Porod volume (nm^3)	86.853	88.679
- χ^2 [total estimate from GNOM]	0.7596	0.7712
- Mass estimate based on volume (Da)	57902	59119
- Mass estimate base on Size & Shape tool in ATSAS 3.0.4 (Da)	59562	64695

(d) Software employed for SAXS data reduction, analysis and interpretation

SAXS data reduction and data processing	EDNA and Primus (ATSAS 3.0.4)
Shape/bead modelling	DAMMIF(ATSAS 3.0.4)
Atomic structure modelling	EOM (ATSAS 3.0.4)
3D graphic representation	UCSF Chimera 1.15
Missing sequence modelling	MODELLER

(e) Shape model-fitting results and rigid-body atomistic modelling

	PTG-PP1	PTG-PP1 + β-cyclodextrin
**DAMMIF**	*Default parameters, 20 calculation runs*	
- q-range (nm^{-1})	0.0051-3.47	0.0063-2.23
- Symmetry	P1	P1
- Normalized spatial discrepancy, σ	0.918, 0.084	0.948, 0.067
- χ^2 range values for the fitting	1.039-1.043	1.046-1.062
- Resolution (from SASRES, in Å)	40 ± 3	41 ± 3
**EOM**	*10,000 models in initial ensemble, random coil models, constant subtraction allowed, 5 independent replica*	
- PDB id for rigid bodies	PTG: 7QM2; PP1: 7QM2	PTG: 7QM2; PP1: 7QM2
- Rigid bodies (residue numbers)	PTG (83-102, 111-128, 133-259). PP1 (7-299)	PTG (83-102, 111-128, 133-259). PP1 (7-299)
- Position of rigid bodies in the space during simulation	PTG(83-102): fixed; PTG(133-259): free; PTG(111-128): fixed. PP1(7-299): fixed	PTG(83-102): fixed; PTG(133-259): free; PTG(111-128): fixed. PP1(7-299): fixed
- Missing segments added *via* EOM	PTG: 70-82, 103-110, 128-132. PP1: 300-330	PTG: 70-82, 103-110, 128-132. PP1: 300-330
- χ^2 value for the fitting	1.06	1.9
- CORMAP *P*-values*	0.042	0.032
- Number of representative structures	5	5
- R_{flex} ensemble (%)	87.27	79.75
- R_{flex} pool (%)	85.57	85.23
- R_{σ} ensemble	1.46	1.06
- Average R_g of ensembles (nm)	3.40	3.53
- Average D_{max} of ensembles (nm)	13.50	13.85
- Fraction of representative structures in the ensemble (%)	Equally represented (20%/each)	Equally represented (20%/each)

(f) Small Angle Scattering Biological Data Bank (SASBDB)

	PTG-PP1	PTG-PP1 + β-cyclodextrin
SASBDB ID	SASDNF2	SASDNG2

* CORMAP is a correlation-based goodness-of-fit test for assessing differences between one-dimensional datasets using only data point correlations over the recorded q-range or part of it, independently of error estimates [1]. *P*-values are $0.01 \leq P < 0.05$.

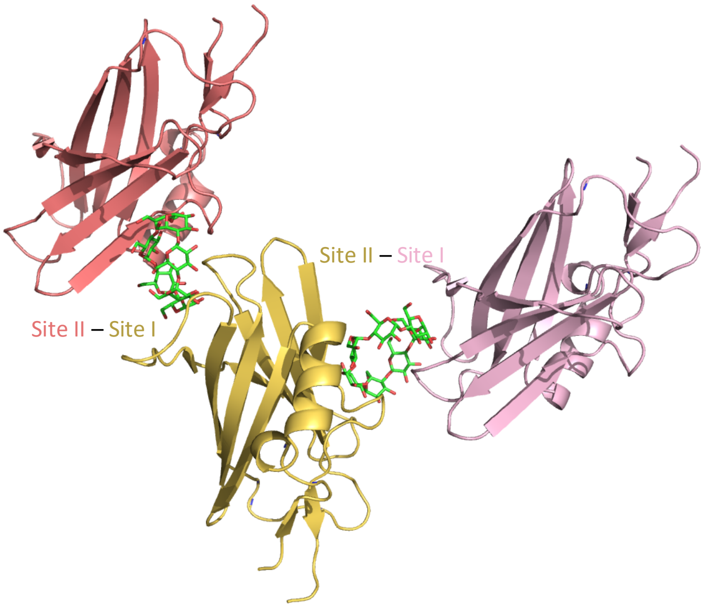

Supplementary Figure 1. β-cyclodextrin (green) locates at the crystallographic interface between PTG chains (yellow, salmon and violet)

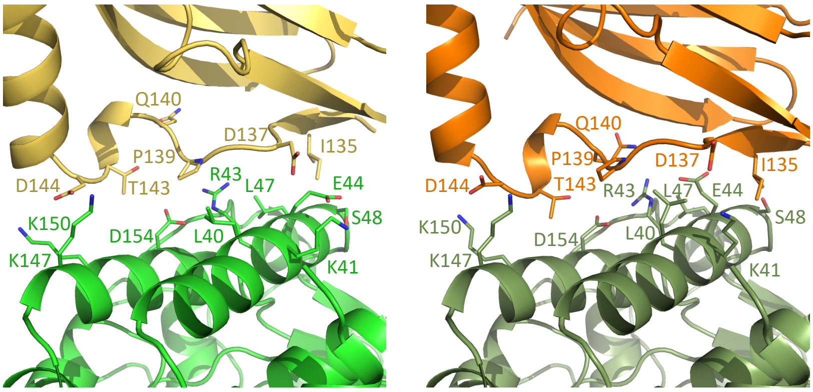

Supplementary Figure 2. Binding interfaces between PP1 (light and dark green) and PTG CBM21 (yellow and orange) are slightly different in the two dimers in the asymmetric unit.

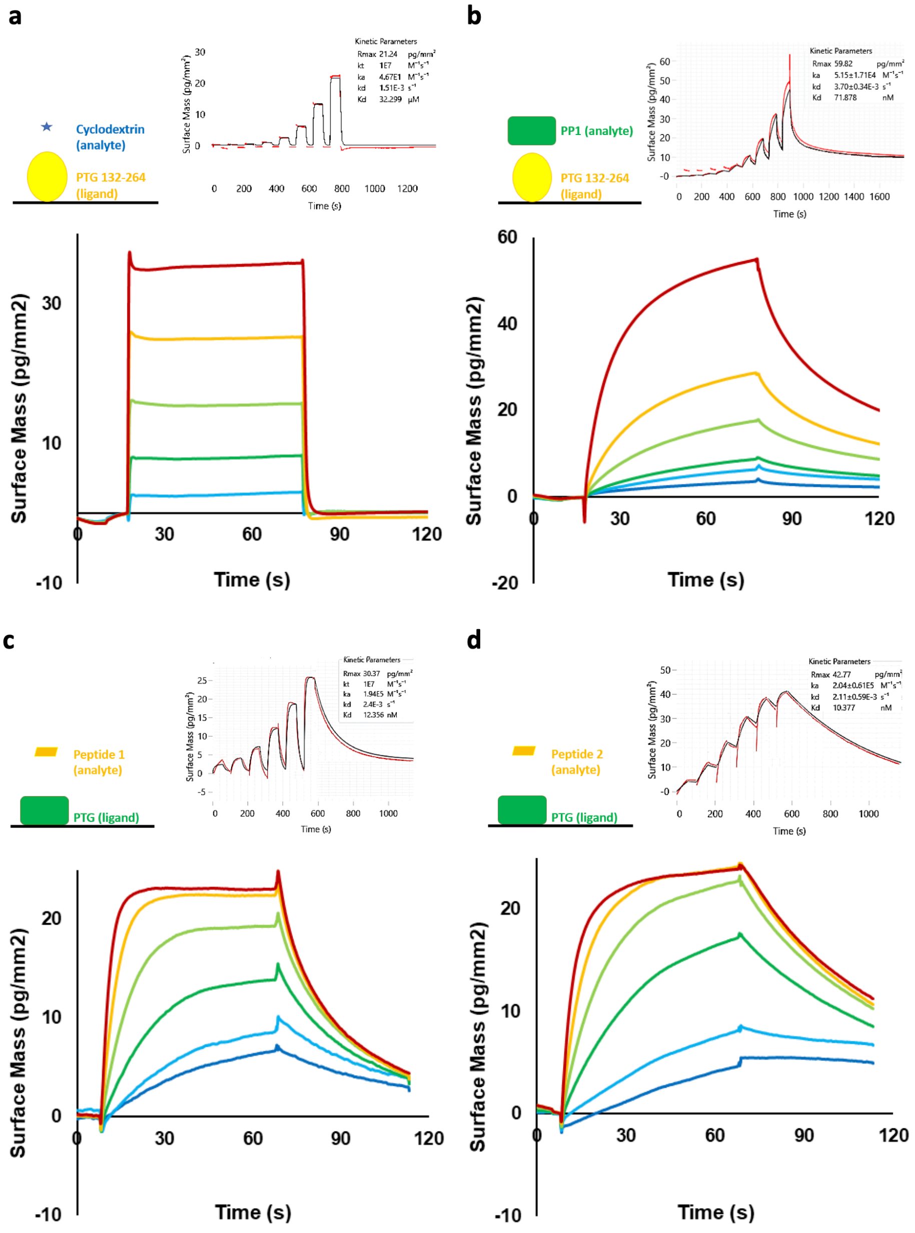

Supplementary Figure 3: Quantitative binding kinetics by GCI. For each experiment, in the top right panel a schematic representation of the experiment is reported, while in the top left panel the experimental sensograms (in red) overlaid with the model fitting curves (in black) are shown. The adjusted raw data are reported in the bottom panel. All curves were blank subtracted. Experiments were performed in triplicate. All the quality assessments (i.e., χ^2 shown in table S2, parameters errors and residual plots were acceptable, the sensograms had sufficient curvatures and the kinetic constant k_{off} were within the measurable range) were fulfilled.

a. PTG 132-264 versus cyclodextrin. Response curves of 1:3 serial dilutions of cyclodextrin (500 µM - 6 µM range) on a cell coated with 1800 pg/mm^2 of PTG 132-264.
b. PP1 PTG 132-264 versus PP1. Response curves of 1:2 serial dilutions of PP1 (400 nM - 12.5 nM range) on a cell coated with 1000 pg/mm^2 of PTG 132-264.
c. PP1 versus peptide 1. Response curves of 1:2 serial dilutions of peptide 1 (300 nM – 9 nM range) on a cell coated with 3600 pg/mm^2 of PP1.
d. PP1 versus peptide 2. Response curves of 1:2 serial dilutions of peptide 2 (300 nM – 9 nM range) on a cell coated with 1800 pg/mm^2 of PP1.

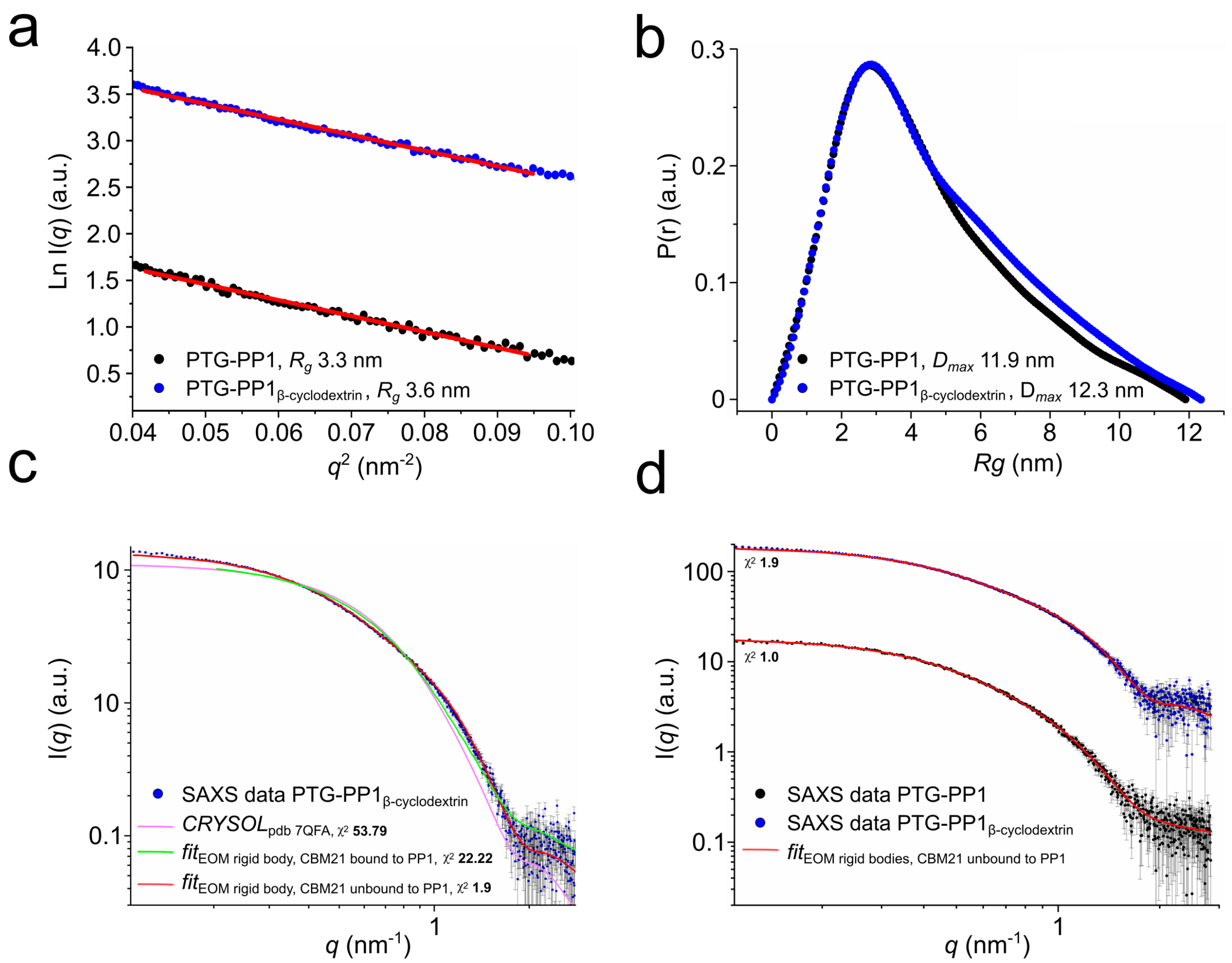

Supplementary Figure 4. SAXS data analysis.

a. Guinier region for the two experimental samples and linear regression (red line) for R_g evaluation.

b. Distance distribution function, P(*r*), of binary (in black) and ternary (in blue) complexes.

c. SAXS curve of the ternary complex and theoretical fitting curves obtained from CRYSOL tool using the X-ray structure of the ternary complex solved in this study (light blue line), EOM tool using a ternary complex with CBM21 bound to PP1 (green line) and EOM tool using a ternary complex with CBM21 unbound to PP1 (red line). Data are shown as log I(*q*) as a function of log (*q*) to enhance the differences in the fitting curves. SAXS curves were obtained through averaging of buffer-background subtracted frames across the entire elution traces of the SEC-SAXS experiments (approximately n = 50 frames averaged, see Supplementary Table 4). Error bars represent an estimate of the experimental error, σ, on the intensity recorded for each value of *q* assigned by data reduction software [2-3].

d. SAXS curves of binary and ternary complexes overlaid with EOM fitting curves. Data are shown as log I(*q*) as a function of log (*q*) to enhance the differences in the fitting curves.

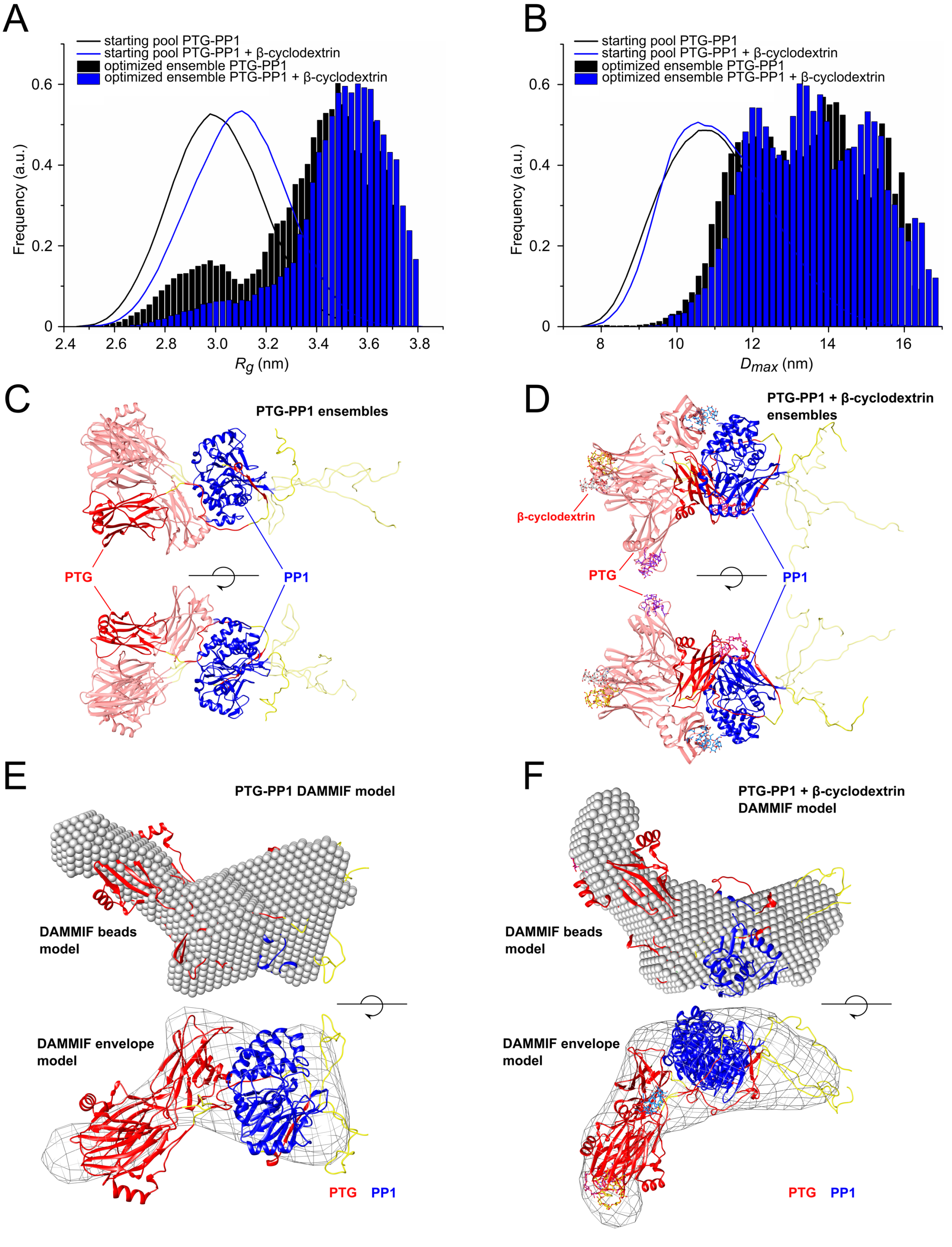

Supplementary Figure 5. Rigid body analysis.

Shown are size distributions, R_g **(a)** and D_{max} **(b)**, of PTG-PP1 and PTG-PP1 + β-cyclodextrin providing qualitative assessment through direct comparison of the distributions of the optimized ensembles (black and blue columns for apo and β-cyclodextrin-bound complex, respectively) and the pools (black and blue lines for apo and β-cyclodextrin-bound complex, respectively).

In panels **(c)** and **(d)**, illustrative 3D structures obtained after EOM modelling describing the PTG-PP1 and PTG-PP1 + β-cyclodextrin ensembles, respectively. In blue, PP1 XRD structure obtained in this study (residues 7-299) and used as rigid body; in red PTG XRD structure obtained in this study (residues 83-102, 133-259 and 111-128) and used as rigid bodies; in yellow missing segments modelled by EOM.

In panels **(e)** and **(f)**, *ab initio* DAMMIF models for PTG-PP1 complex and PTG-PP1 + β-cyclodextrin, respectively, superposed with representative EOM ensembles. DAMMIF structures are shown as beads and volume models.

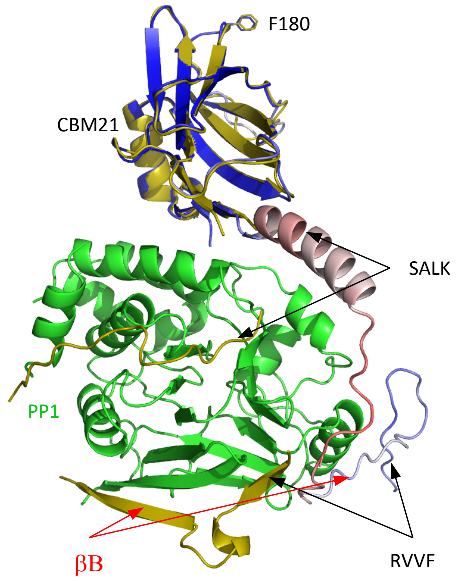

Supplementary Figure 6. Comparison of experimental and AlphaFold-predicted PTG structures. AlphaFold-predicted PTG (https://www.alphafold.ebi.ac.uk/entry/Q9UQK1, colored blue to red according to the confidence score) has been superposed to the crystal structure of PTG (yellow) in complex with PP1 (green). The agreement between model and structure is very good in the CBM21 region, while poor in the remaining regions.

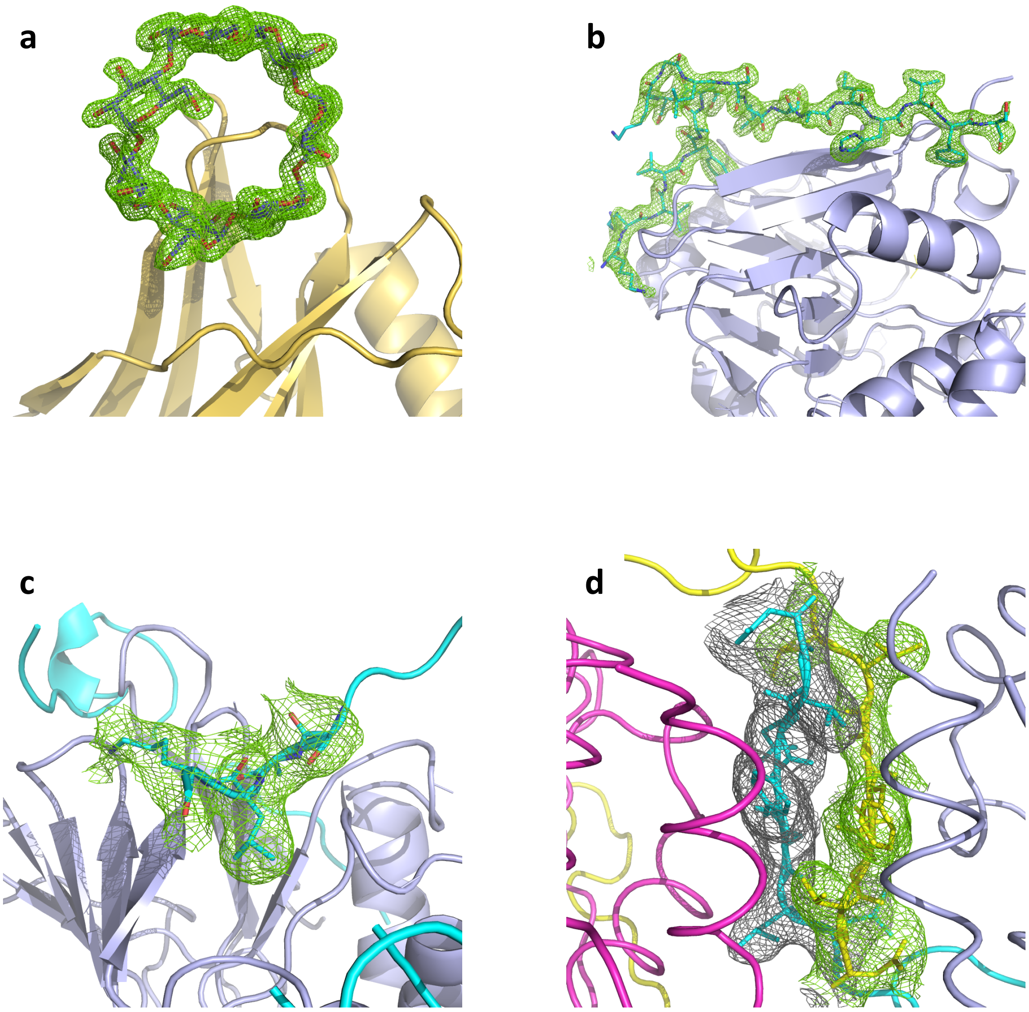

Supplementary Figure 7. Representative F_o-F_c polder OMIT maps [4] contoured at 3 σ.

a. β-ciclodextrin bound to the PTG-CBM21 (yellow) in PDB 7QF7.
b. PTG RVVF peptide (cyan) bound to PP1 (violet) in PDB 7QFB.
c. PTG SALK region (cyan) bound to PP1 (violet) in PDB 7QM2.
d. PTG dimerization strands (yellow and cyan, PP1 in magenta and violet) in PDB 7QM2.

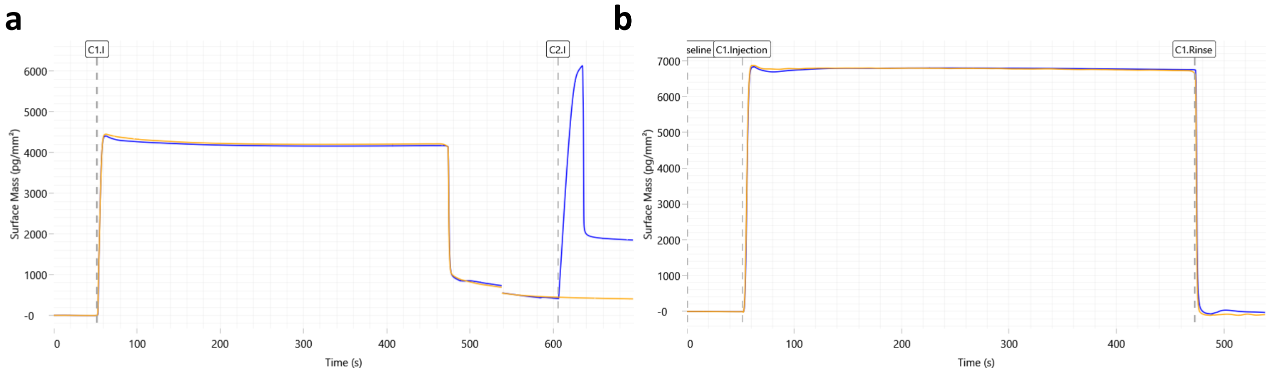

Supplementary Figure 8. PTG immobilization by amine coupling for cyclodextrin kinetics: a) activation with EDC/NHS mix injected for 420 s, followed by injection of PTG (20 ug/ml in buffer acetate pH 5.0) for 30 s; b) passivation with ethanolamine injected for 420s. Reference channel is in yellow, in blue the channel carrying PTG. For all the immobilization procedure a flux of 10 ul/sec was employed. The surface was prepared once for all the performed experiments.

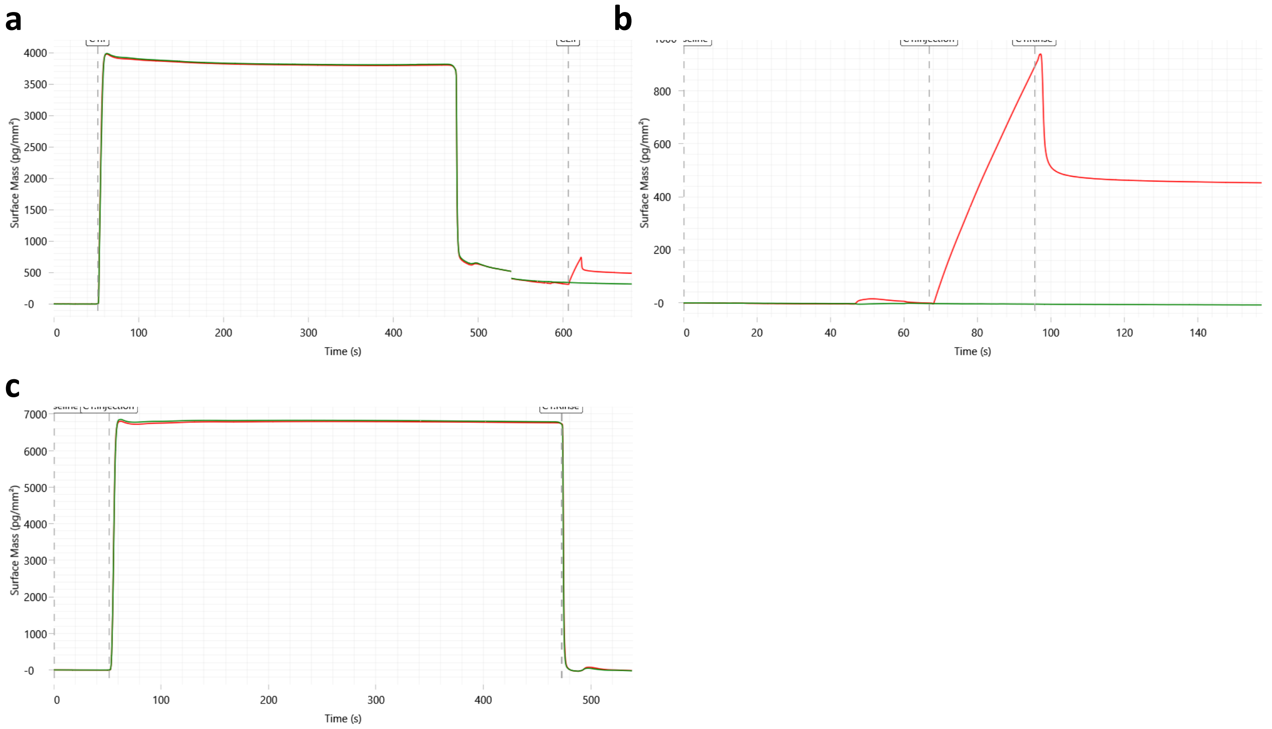

Supplementary Figure 9. PTG immobilization by amine coupling for PP1 kinetics: a) activation with EDC/NHS mix injected for 420 s, followed by injection of PTG (20 ug/ml in buffer acetate pH 5.0) for 15s; b) injection of PTG (20 ug/ml in buffer acetate pH 5.0) for 30s to reach the desired density to analyse PP1 kinetics; c) passivation with ethanolamine injected for 420 s. Reference channel is in green, in red the channel carrying PTG. For all the immobilization procedure a flux of 10 ul/sec was employed. The surface was prepared once for all the performed experiments.

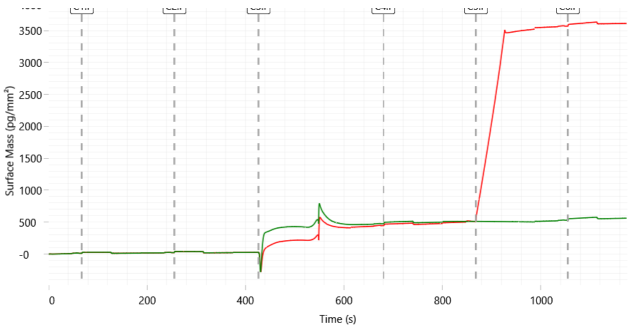

Supplementary Figure 10. PP1 capture by NTA surface for Peptide1 kinetics: After two startups, activation with 0.5mM $NiCl_2$ injected for 120 s (C3.1) followed by injection of PP1 (5 ug/ml in running buffer) for 60s (C5.1) to reach the desired density to analyse Peptide1 kinetics. Reference channel is in green, in red the channel carrying PP1. For the capture procedure a flux of 10 ul/sec was employed. Each experiment was followed by a regeneration procedure injecting 350 mM EDTA for 420s the regeneration of the NTA surface. At each experiment the captured ligand level was constant, i.e. 3600±97, taking into account the experimental error in diluting the ligand solution.

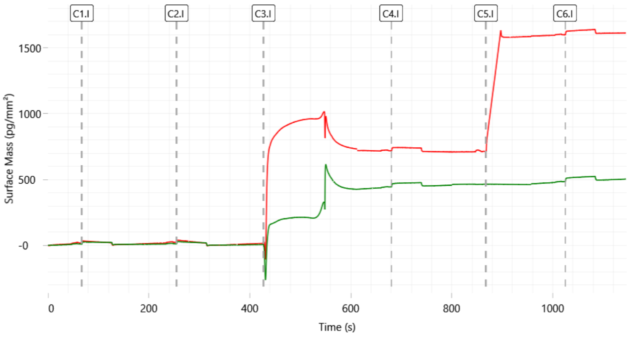

Supplementary Figure 11. PP1 capture by NTA surface for Peptide2 kinetics: After two startups, activation with 0.5mM $NiCl_2$ injected for 120 s (C3.1) followed by injection of PP1 (5 ug/ml in running buffer) for 30s (C5.1) to reach the desired density to analyse Peptide2 kinetics. Reference channel is in green, in red the channel carrying PP1. For the capture procedure a flux of 10 ul/sec was employed. Each experiment was followed by a regeneration procedure injecting 350 mM EDTA for 420s the regeneration of the NTA surface. At each experiment the captured ligand level was constant, i.e. 1800±52, taking into account the experimental error in diluting the ligand solution.

SUPPLEMENTARY REFERENCES

1. Franke D, Jeffries CM, Svergun DI. Correlation Map, a goodness-of-fit test for one-dimensional X-ray scattering spectra. Nat. Methods. 2015, 12:419-22.
2. Svergun DI, Pedersen JS. Propagating errors in small-angle scattering data treatment. J. Appl. Cryst. 1994, 27:241-8.
3. Larsen AH, Pedersen MC. Experimental noise in small-angle scattering can be assessed using the Bayesian indirect Fourier transformation. J. Appl. Cryst. 2021, 54:1281-89.
4. Liebschner D., Afonine P.V., Moriarty N.W., Poon B.K., Sobolev O.V., Terwilliger T.C., and Adams P.D. (2017) Polder maps: improving OMIT maps by excluding bulk solvent. *Acta Crystallogr. D Struct. Biol. 73*, 148-57.
