# Supplementary material for: Molecular architecture of the glycogen- committed PP1/PTG holoenzyme
Source: Nat Commun. 2022 Oct 19;13:6199. doi: 10.1038/s41467-022-33693-z (PMC9582199; doi:10.1038/s41467-022-33693-z)
Supplement: Supplementary file 2 — Reporting Summary [file 41467_2022_33693_MOESM2_ESM.pdf]

Corresponding author(s): Paola Storici and Graziano Lolli

Last updated by author(s): Sep 12, 2022

Reporting Summary

Nature Portfolio wishes to improve the reproducibility of the work that we publish. This form provides structure for consistency and transparency in reporting. For further information on Nature Portfolio policies, see our Editorial Policies and the Editorial Policy Checklist.

Statistics

For all statistical analyses, confirm that the following items are present in the figure legend, table legend, main text, or Methods section.

n/a	Confirmed	
☐	☒	The exact sample size (*n*) for each experimental group/condition, given as a discrete number and unit of measurement
☐	☒	A statement on whether measurements were taken from distinct samples or whether the same sample was measured repeatedly
☐	☒	The statistical test(s) used AND whether they are one- or two-sided<br>*Only common tests should be described solely by name; describe more complex techniques in the Methods section.*
☒	☐	A description of all covariates tested
☒	☐	A description of any assumptions or corrections, such as tests of normality and adjustment for multiple comparisons
☐	☒	A full description of the statistical parameters including central tendency (e.g. means) or other basic estimates (e.g. regression coefficient) AND variation (e.g. standard deviation) or associated estimates of uncertainty (e.g. confidence intervals)
☐	☒	For null hypothesis testing, the test statistic (e.g. *F*, *t*, *r*) with confidence intervals, effect sizes, degrees of freedom and *P* value noted<br>*Give P values as exact values whenever suitable.*
☒	☐	For Bayesian analysis, information on the choice of priors and Markov chain Monte Carlo settings
☒	☐	For hierarchical and complex designs, identification of the appropriate level for tests and full reporting of outcomes
☒	☐	Estimates of effect sizes (e.g. Cohen's *d*, Pearson's *r*), indicating how they were calculated

Our web collection on statistics for biologists contains articles on many of the points above.

Software and code

Policy information about availability of computer code

Data collection	ITC200 control system 1.24.2
Data analysis	Global Phasing autoPROC pipeline 1.0.5, Coot 0.8.9.2, ATSAS 3.0.4 package, CCP4 suite 7.0.078, Phenix 1.11.1, Wave control 4.4.5, ORIGIN7 7.0552, UCSF CHIMERA 1.15, OriginPro 9.0

For manuscripts utilizing custom algorithms or software that are central to the research but not yet described in published literature, software must be made available to editors and reviewers. We strongly encourage code deposition in a community repository (e.g. GitHub). See the Nature Portfolio guidelines for submitting code & software for further information.

Data

Policy information about availability of data

All manuscripts must include a data availability statement. This statement should provide the following information, where applicable:

- Accession codes, unique identifiers, or web links for publicly available datasets
- A description of any restrictions on data availability
- For clinical datasets or third party data, please ensure that the statement adheres to our policy

Data and structures have been deposited in the PDB database under accession codes: 7QF7 [https://doi.org/10.2210/pdb7QF7/pdb], 7QFA [https://doi.org/10.2210/pdb7QFA/pdb], 7QFB [https://doi.org/10.2210/pdb7QFB/pdb] and 7QM2 [https://doi.org/10.2210/pdb7QM2/pdb].
SAXS data have been deposited in the Small Angle Scattering Biological Data Bank (SASBDB) under accession numbers SASDNF2 [https://www.sasbdb.org/data/SASDNF2] and SASDNG2 [https://www.sasbdb.org/data/SASDNG2].

Field-specific reporting

Please select the one below that is the best fit for your research. If you are not sure, read the appropriate sections before making your selection.

☒ Life sciences ☐ Behavioural & social sciences ☐ Ecological, evolutionary & environmental sciences

For a reference copy of the document with all sections, see nature.com/documents/nr-reporting-summary-flat.pdf

Life sciences study design

All studies must disclose on these points even when the disclosure is negative.

Sample size	Number of replicas, three for GCI and two for ITC, has been chosen in order to test the reliability of single values (kon, kof, Kd for GCI, KA, deltaH and n for ITC).
Data exclusions	Three points were excluded in the ITC titrations over 120 total due to poor fitting, possibly ascribable to air bubbles.
Replication	Experiments were replicated three times for GCI and two times for ITC.
Randomization	Randomization not relevant. Experiments do not involve comparison between groups of measurements. A control experiment is performed in ITC for background subtraction purpose only.
Blinding	Blinding not relevant. Due to the complex nature of the GCI and ITC measurements, it is very unlikely that the measurements are affected by observer bias. This is especially true for the presented experiments were the difference between significant and non-significant events is clearcut, as displayed by the comparison of sample vs control measurements.

Reporting for specific materials, systems and methods

We require information from authors about some types of materials, experimental systems and methods used in many studies. Here, indicate whether each material, system or method listed is relevant to your study. If you are not sure if a list item applies to your research, read the appropriate section before selecting a response.

Materials & experimental systems

n/a	Involved in the study	
☒	☐	Antibodies
☒	☐	Eukaryotic cell lines
☒	☐	Palaeontology and archaeology
☒	☐	Animals and other organisms
☒	☐	Human research participants
☒	☐	Clinical data
☒	☐	Dual use research of concern

Methods

n/a	Involved in the study	
☒	☐	ChIP-seq
☒	☐	Flow cytometry
☒	☐	MRI-based neuroimaging
